# Supplementary material for: MUC1-C intersects chronic inflammation with epigenetic reprogramming by regulating the set1a compass complex in cancer progression
Source: Commun Biol. 2023 Oct 11;6:1030. doi: 10.1038/s42003-023-05395-9 (PMC10567710; doi:10.1038/s42003-023-05395-9)
Supplement: Supplementary file 2 — Supplementary Information [file 42003_2023_5395_MOESM2_ESM.pdf]

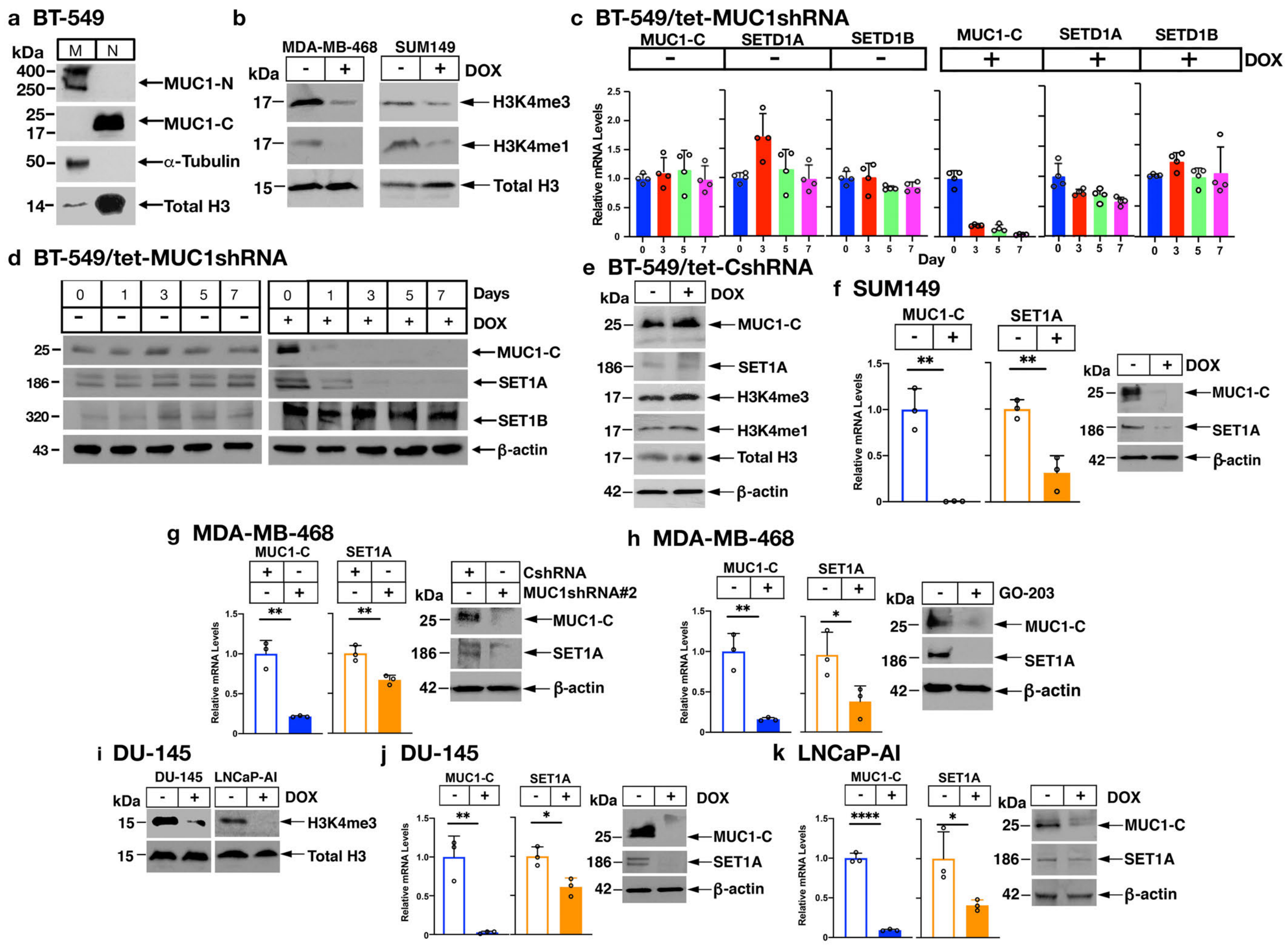

Supplemental Figure S1. Targeting MUC1-C suppresses SET1A and H3K4 trimethylation in TNBC and CRPC cells. a. Lysates of BT-549 cell membrane and nuclear fractions were immunoblotted with antibodies against the indicated proteins. b. Lysates from MDA-MB-468/tet-MUC1shRNA and SUM149/tet-MUC1shRNA cells treated with vehicle or DOX for 7 days were immunoblotted with antibodies against the indicated proteins. **c and d.** BT-549/tet-MUC1shRNA cells treated with vehicle or DOX for the indicated days were analyzed for MUC1-C, SET1A and SET1B mRNA levels by qRT-PCR. The results (mean±SD of 3 determinations) are expressed as relative mRNA levels compared to that obtained for day 0 cells (assigned a value of 1) (**c**). Lysates were immunoblotted with antibodies against the indicated proteins (**d**). **e.** BT-549/tet-CshRNA cells treated with vehicle or DOX for 7 days were immunoblotted with antibodies against the indicated proteins. **f.** SUM149/tet-MUC1shRNA cells treated with vehicle or DOX for 7 days were analyzed for MUC1-C and SET1A mRNA levels by qRT-PCR. The results (mean±SD of 3 determinations) are expressed as relative mRNA levels compared to that obtained for vehicle-treated cells (assigned a value of 1) (left).

Lysates were immunoblotted with antibodies against the indicated proteins (right). **g.** MDA-MB-468/CshRNA and MDA-MB-468/MUC1shRNA#2 cells were analyzed for MUC1-C and SET1A mRNA levels by qRT-PCR. The results (mean±SD of 3 determinations) are expressed as relative mRNA levels compared to that obtained for CshRNA cells (assigned a value of 1) (left). Lysates were immunoblotted with antibodies against the indicated proteins (right). **h.** MDA-MB-468 cells treated with vehicle or 5 μM GO-203 for 48 h were analyzed for MUC1-C and SET1A mRNA levels by qRT-PCR. The results (mean±SD of 3 determinations) are expressed as relative mRNA levels compared to that obtained for vehicle treated cells (assigned a value of 1) (left). Lysates were immunoblotted with antibodies against the indicated proteins (right). **i.** Lysates from DU-145/tet-MUC1shRNA (left) and LNCAP-AI/tet-MUC1shRNA (right) cells treated with vehicle or DOX for 7 days immunoblotted with antibodies against the indicated proteins. **j and k.** DU-145/tet-MUC1shRNA (**j**) and LNCaP-AI/tet-MUC1shRNA (**k**) cells treated with vehicle or DOX for 7 days were analyzed for MUC1-C and SET1A mRNA levels by qRT-PCR. The results (mean±SD of 3 determinations) are expressed as relative mRNA levels compared to that obtained for vehicle-treated cells (assigned a value of 1)(left). Lysates were immunoblotted with antibodies against the indicated proteins (right).

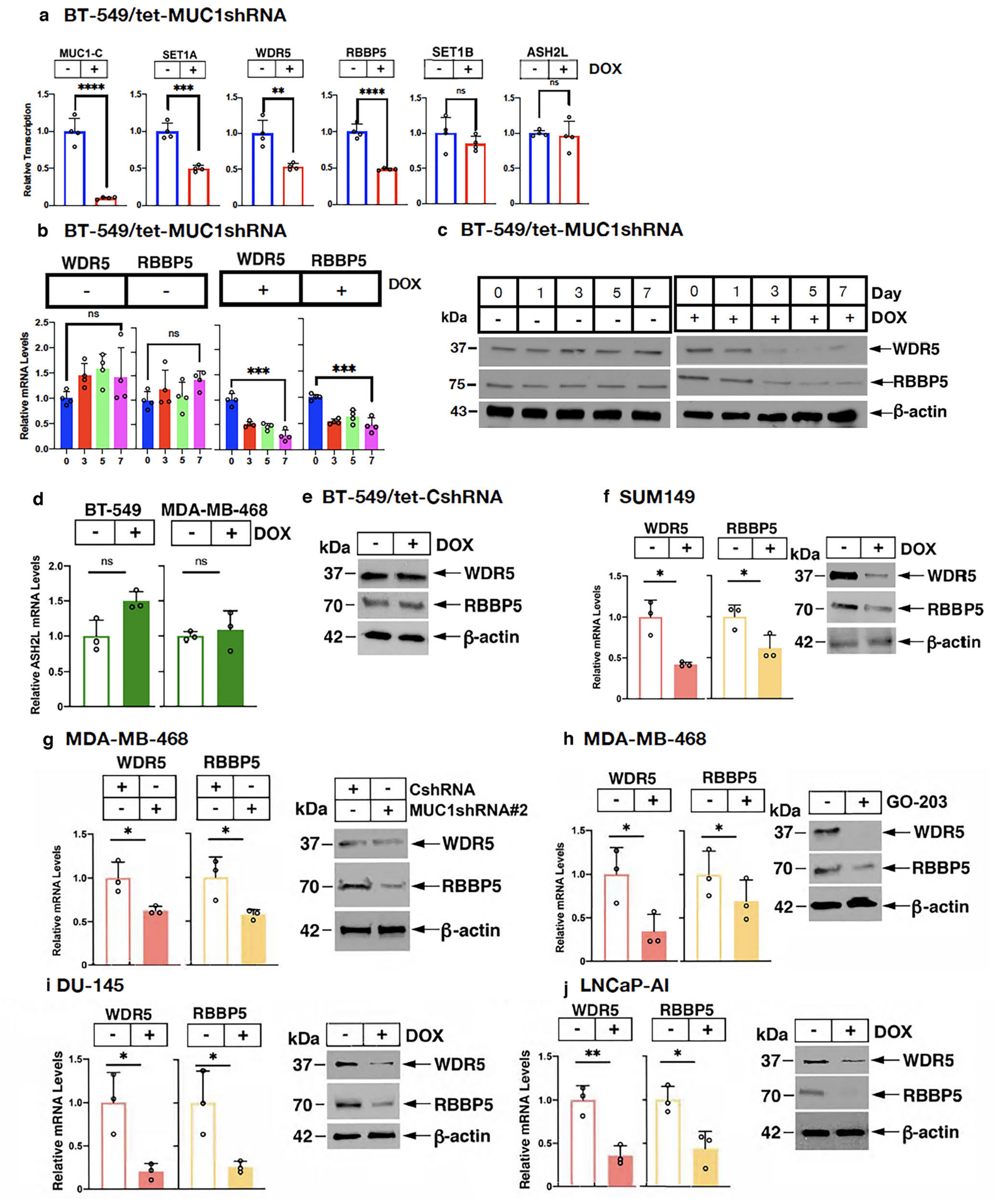

Supplemental Figure S2. Targeting MUC1-C suppresses WDR5 and RBBP5 expression in TNBC and CRPC cancer cells. a. BT-549/tet-MUC1shRNA cells treated with vehicle or DOX for 7 days were analyzed for transcription of the indicated genes. The results (mean±SD of 4

determinations) are expressed as relative levels compared to that obtained for vehicle-treated cells (assigned a value of 1). **b and c.** BT-549/tet-MUC1shRNA cells treated with vehicle or DOX for the indicated days were analyzed for WDR5 and RBBP5 mRNA levels by qRT-PCR. The results (mean±SD of 4 determinations) are expressed as relative mRNA levels compared to that obtained for vehicle-treated cells (assigned a value of 1)(**b**). Lysates were immunoblotted with antibodies against the indicated proteins (**c**). **d.** BT-549/tet-MUC1shRNA (left) and MDA-MB-468/tet-MUC1shRNA (right) cells treated with vehicle or DOX for 7 days were analyzed for ASH2L mRNA levels by qRT-PCR. The results (mean±SD of 3 determinations) are expressed as relative MUC1-C mRNA levels compared to that obtained for vehicle treated cells (assigned a value of 1). **e.** Lysates from BT-549/tet-CshRNA cells treated with vehicle of DOX for 7 days were immunoblotted with antibodies against the indicated proteins. **f.** SUM149/tet-MUC1shRNA cells treated with vehicle or DOX for 7 days were analyzed for WDR5 and RBBP5 mRNA levels by qRT-PCR. The results (mean±SD of 3 determinations) are expressed as relative mRNA levels compared to that obtained for vehicle-treated cells (assigned a value of 1)(left). Lysates were immunoblotted with antibodies against the indicated proteins (right). **g.** MDA-MB-468/CshRNA and MDA-MB-468/MUC1shRNA#2 cells were analyzed for WDR5 and RBBP5 mRNA levels by qRT-PCR. The results (mean±SD of 3 determinations) are expressed as relative mRNA levels compared to that obtained for CshRNA cells (assigned a value of 1)(left). Lysates were immunoblotted with antibodies against the indicated proteins (right). **h.** MDA-MB-468 cells treated with vehicle or 5 μM GO-203 for 48 h were analyzed for WDR5 and RBBP5 mRNA levels by qRT-PCR. The results (mean±SD of 3 determinations) are expressed as relative mRNA levels compared to that obtained for vehicle-treated cells (assigned a value of 1)(left). Lysates were immunoblotted with antibodies against the indicated proteins (right). **i and j.** DU-145/tet-MUC1shRNA (**i**) and LNCAP-AI/tet-MUC1shRNA (**j**) cells treated with vehicle or DOX for 7 days were analyzed for WDR5 and RBBP5 mRNA levels by qRT-PCR. The results (mean±SD of 3 determinations) are expressed as relative MUC1-C mRNA levels compared to that obtained for vehicle-treated cells (assigned a value of 1)(left). Lysates were immunoblotted with antibodies against the indicated proteins (right).

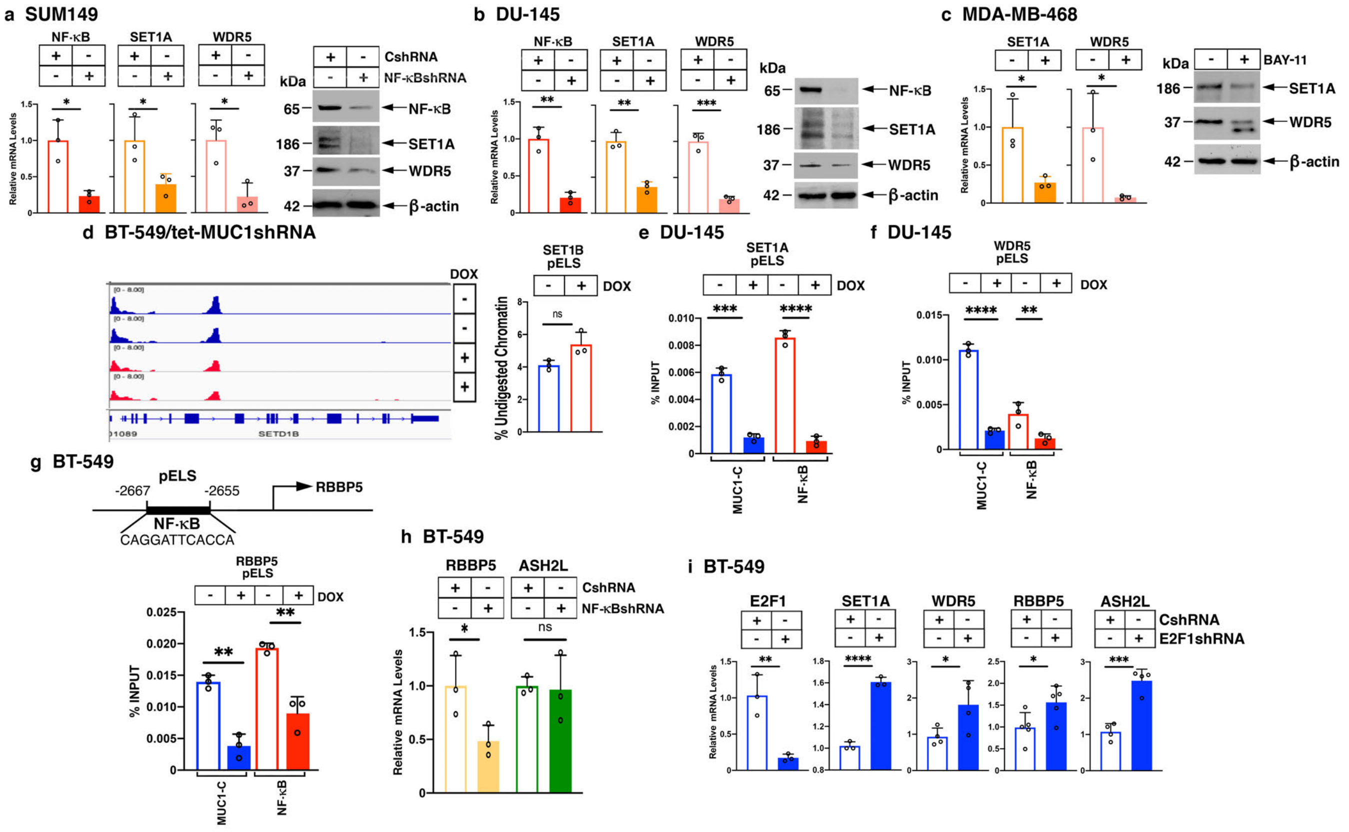

Supplemental Figure S3. Targeting NF-κB suppresses *SET1A* and *WDR5* expression. a and b. SUM149 (**a**) and DU-145 (**b**) cells expressing a CshRNA or NF-κBshRNA were analyzed for NF-κB p65, SET1A and WDR5 mRNA levels by qRT-PCR. The results (mean±SD of 3 determinations) are expressed as relative mRNA levels compared to that obtained for CshRNA cells (assigned a value of 1)(left). Lysates were immunoblotted with antibodies against the indicated proteins (right). **c.** MDA-MB-468 cells treated with vehicle or 5 μM BAY-11 for 16 h were analyzed for SET1 and WDR5 mRNA levels by qRT-PCR. The results (mean±SD of 3 determinations) are expressed as relative mRNA levels compared to that obtained for vehicle treated cells (assigned a value of 1)(left). Lysates were immunoblotted with antibodies against the indicated proteins (right). **d.** Genome browser snapshot of ATAC-seq data from the *SET1B* gene in BT-549/tet-MUC1shRNA cells treated with vehicle or DOX for 7 days (left). (left). Chromatin was analyzed for accessibility by nuclease digestion (right). The results are expressed as % undigested chromatin (mean±SD and individual values). **e and f.** Soluble chromatin from DU-145/tet-MUC1shRNA cells treated with vehicle or DOX for 7 days was precipitated with anti-MUC1-C and anti-NF-κB p65. The DNA samples were amplified by qPCR with primers for the *SET1A* (**e**) and *WDR5* (**f**) pELS regions. The results (mean±SD of 3 determinations) are expressed as percent input. **g.** Scheme of *RBBP5* with highlighting of the indicated NF-κB binding motif. Soluble chromatin from BT-549/tet-MUC1shRNA cells

treated with vehicle or DOX for 7 days was precipitated with anti-MUC1-C and anti-NF-κB p65. The DNA samples were amplified by qPCR with primers for the *RBBP5* pELS region. The results (mean±SD of 3 determinations) are expressed as percent input. **h.** BT-549/CshRNA and BT-549/NF-κBshRNA cells were analyzed for RBBP5 (left) and ASH2L (right) mRNA levels by qRT-PCR. The results (mean±SD of 3 determinations) are expressed as relative mRNA levels compared to that obtained for CshRNA cells (assigned a value of 1). **i.** BT-549/CshRNA and BT-549/E2F1shRNA cells were analyzed for the indicated mRNA levels by qRT-PCR. The results (mean±SD of 3 determinations) are expressed as relative mRNA levels compared to that obtained for CshRNA cells (assigned a value of 1).

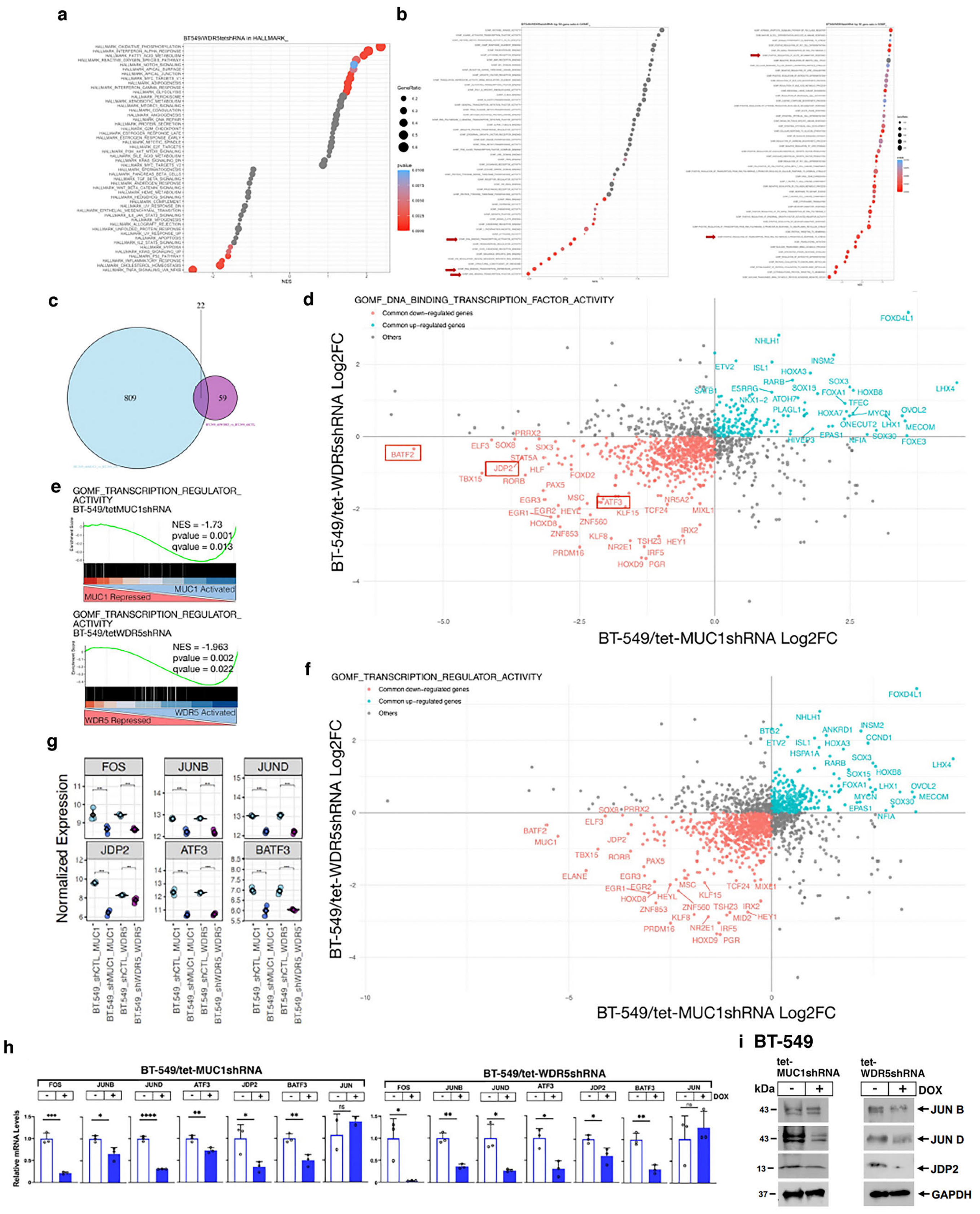

Supplemental Figure S4. Effects of WDR5 silencing on gene activation and repression. a. RNA-seq was performed on BT-549/tet-

WDR5shRNA cells treated with vehicle of DOX for 7 days. GSEA was performed using the HALLMARK gene signatures. **b.** GSEA was performed using the GO Molecular function (GO MF) (left) and GO Biological Processes (GO BP)(right) signatures. **c.** Venn diagram showing overlap of GSEA pathways in MUC1- and WDR5-silenced cells. **d.** Overlap of downregulated (left) and upregulated (right) genes by MUC1 and WDR5 silencing in BT-549 cells using the GOMF DNA BINDING TRANSCRIPTION FACTOR ACTIVITY signature. **e.** GSEA of the MUC1 and WDR5 RNA-seq datasets using the GOMF TRANSCRIPTION REGULATOR ACTIVITY gene signature. **f.** Overlap of activated and repressed genes in MUC1- and WDR5-silenced cells using the GOMF TRANSCRIPTION REGULATOR ACTIVITY gene signature. **g.** Candidate AP-1 genes identified from the RNA-seq data. **h and i.** BT-549/tet-MUC1shRNA (left) and BT-549/tet-WDR5shRNA (right) cells treated with vehicle or DOX for 7 days were analyzed for the indicated mRNA levels by qRT-PCR (**h**). The results (mean±SD of 3 determinations) are expressed as relative mRNA levels compared to that obtained for vehicle treated cells (assigned a value of 1). Lysates were immunoblotted with antibodies against the indicated proteins (**i**).

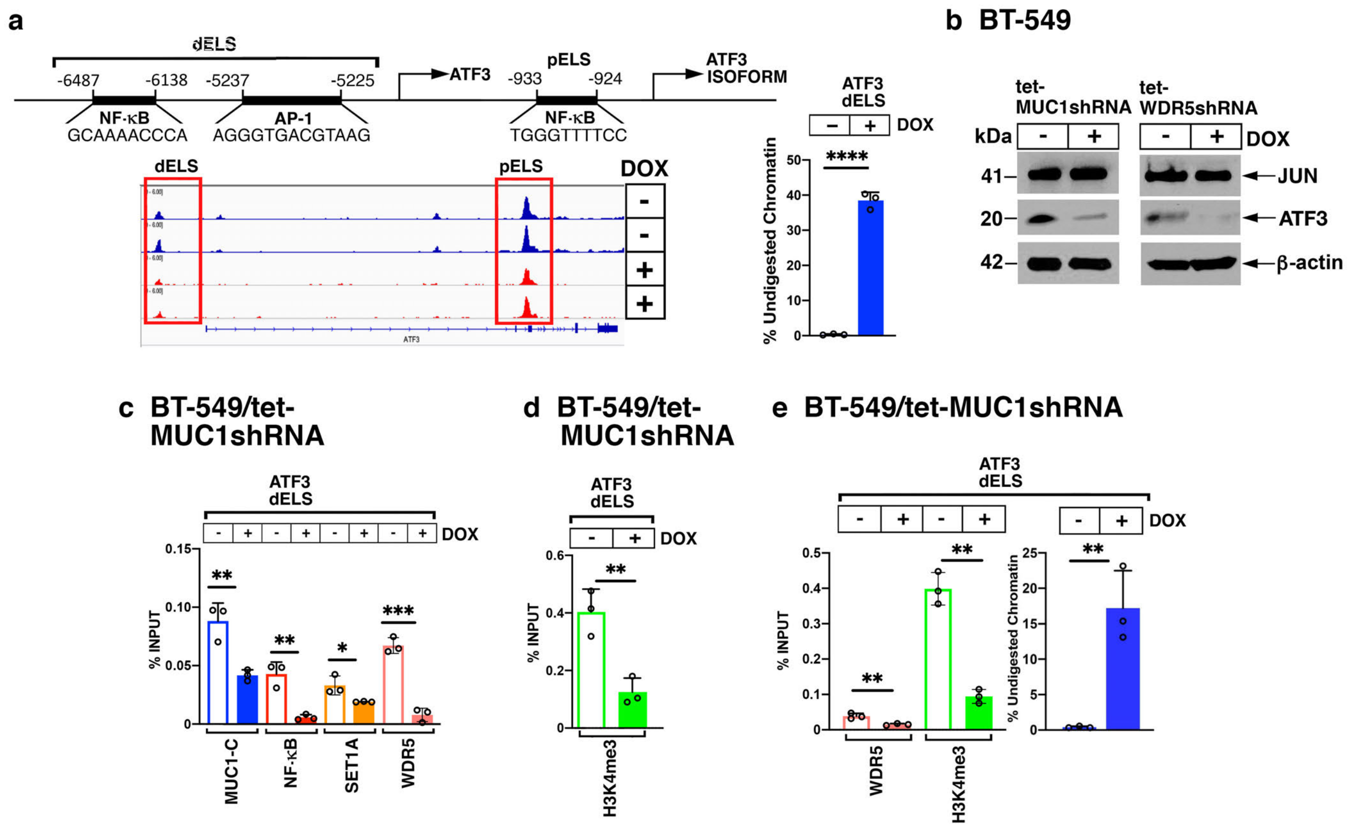

Supplemental Figure S5. MUC1-C and WDR5 are necessary for activation of the *ATF3* gene. a. Genome browser snapshot of ATAC-seq data from the *ATF3* (i) dELS region upstream to the TSS encoding full-length ATF3, and (ii) pELS in the promoter region for an ATF3 isoform in BT-549/tet-MUC1shRNA cells treated with vehicle or DOX for 7 days (left). Chromatin was analyzed for accessibility of the dELS by nuclease digestion (right). The results are expressed as % undigested chromatin (mean±SD and individual values). **b.** Lysates from BT-549/tet-MUC1shRNA (left) and BT-549/tet-WDR5shRNA cells (right) treated with vehicle or DOX for 7 days were immunoblotted with antibodies against the indicated proteins. **c and d.** BT-549/tet-MUC1shRNA cells were treated with vehicle or DOX for 7 days. Soluble chromatin was precipitated with anti-MUC1-C, anti-SET1A and anti-WDR5 (**c**), and anti-H3K4me3 **(d)**. The DNA samples were amplified by qPCR with primers for the *ATF3* dELS. **e.** BT-549/tet-WDR5shRNA cells were treated with vehicle or DOX for 7 days. Soluble chromatin was precipitated with IgG, anti-WDR5 and anti-H3K4me3 (left). The DNA samples were amplified by qPCR with primers for the *ATF3* dELS. The results (mean±SD of 3 determinations) are expressed as percent input. Chromatin was analyzed for accessibility of the *ATF3* dELS by nuclease digestion (right). The results (mean±SD of 3 determinations) are expressed as % undigested chromatin.

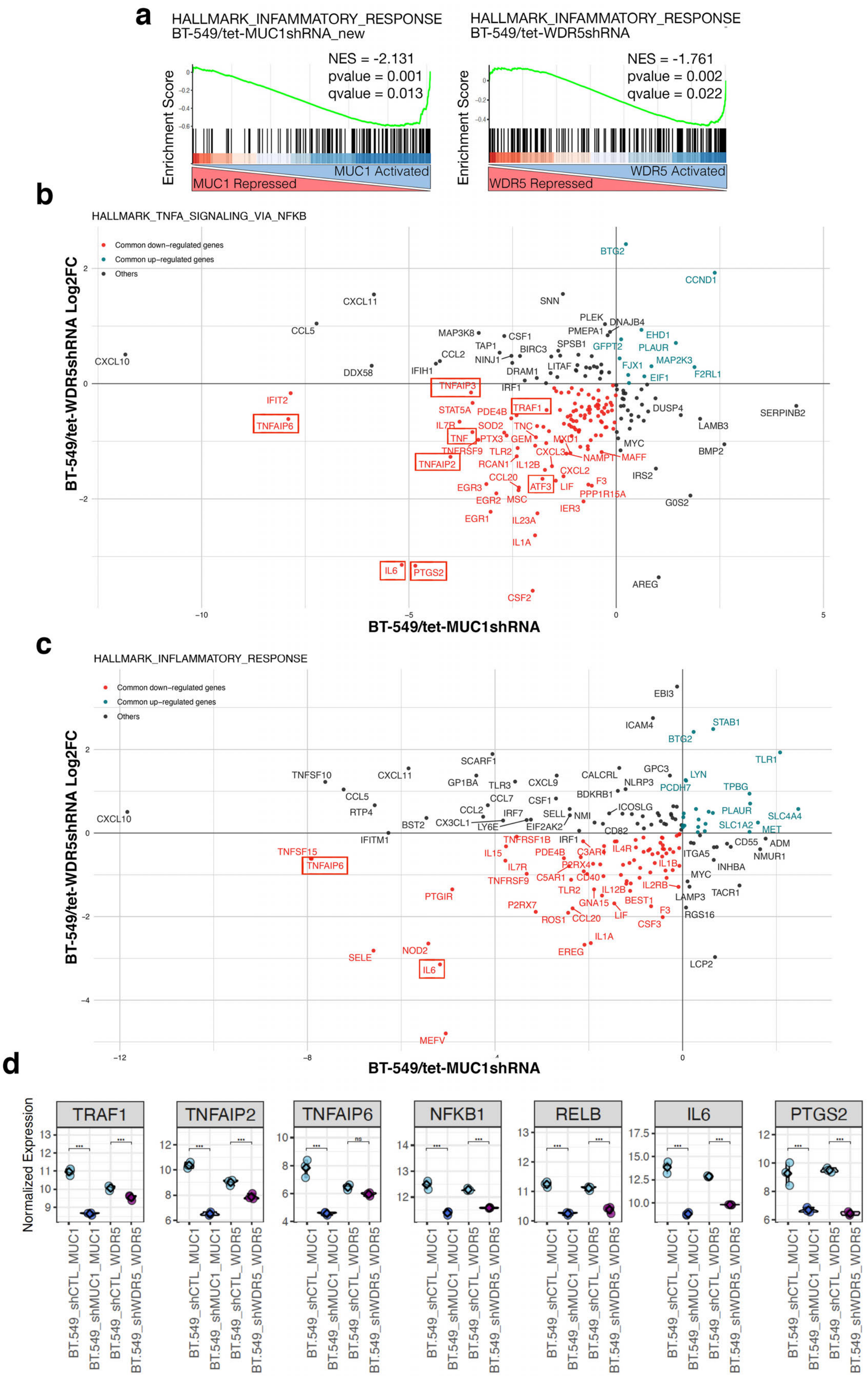
a
HALLMARK_INFAMMATORY_RESPONSE
BT-549/tet-MUC1shRNA_new
NES = -2.131
pvalue = 0.001
qvalue = 0.013
Enrichment Score
MUC1 Activated
MUC1 Repressed
HALLMARK_INFAMMATORY_RESPONSE
BT-549/tet-WDR5shRNA
NES = -1.761
pvalue = 0.002
qvalue = 0.022
Enrichment Score
WDR5 Activated
WDR5 Repressed
b
HALLMARK_TNFA_SIGNALING_VIA_NFKB
Common down−regulated genes
Common up−regulated genes
Others
BT-549/tet-WDR5shRNA Log2FC
BT-549/tet-MUC1shRNA
BTG2
CCND1
CXCL11
SNN
CCL5
CXCL10
DDX58
IFIH1
TNFAIP3
IFIT2
TNFAIP6
TRAF1
TNF
TNFAIP2
ATF3
IL6
PTGS2
CSF2
SERPINB2
AREG
c
HALLMARK_INFLAMMATORY_RESPONSE
Common down−regulated genes
Common up−regulated genes
Others
BT-549/tet-WDR5shRNA Log2FC
BT-549/tet-MUC1shRNA
EBI3
ICAM4
STAB1
BTG2
TLR1
TNFSF10
CXCL11
CXCL10
TNFSF15
TNFAIP6
PTGIR
SELE
NOD2
IL6
MEFV
LCP2
d
Normalized Expression
TRAF1
TNFAIP2
TNFAIP6
NFKB1
RELB
IL6
PTGS2
BT.549_shCTL_MUC1
BT.549_shMUC1_MUC1
BT.549_shCTL_WDR5
BT.549_shWDR5_WDR5

Supplemental Figure S6. MUC1 and WDR5 regulate common sets of genes that promote chronic inflammation. a. GSEA of the RNA-seq datasets from MUC1- (left) and WDR5- (right) silenced cells using the HALLMARK INFLAMMATORY RESPONSE gene signature. **b and c.** Overlap of activated and repressed genes in MUC1- and WDR5-silenced cells using HALLMARK TNFA SIGNALING VIA NFKB (**b**) and HALLMARK INFLAMMATORY RESPONSE (**c**) gene signatures. **d.** Candidate plots showing common inflammatory response genes from the RNA-seq data.

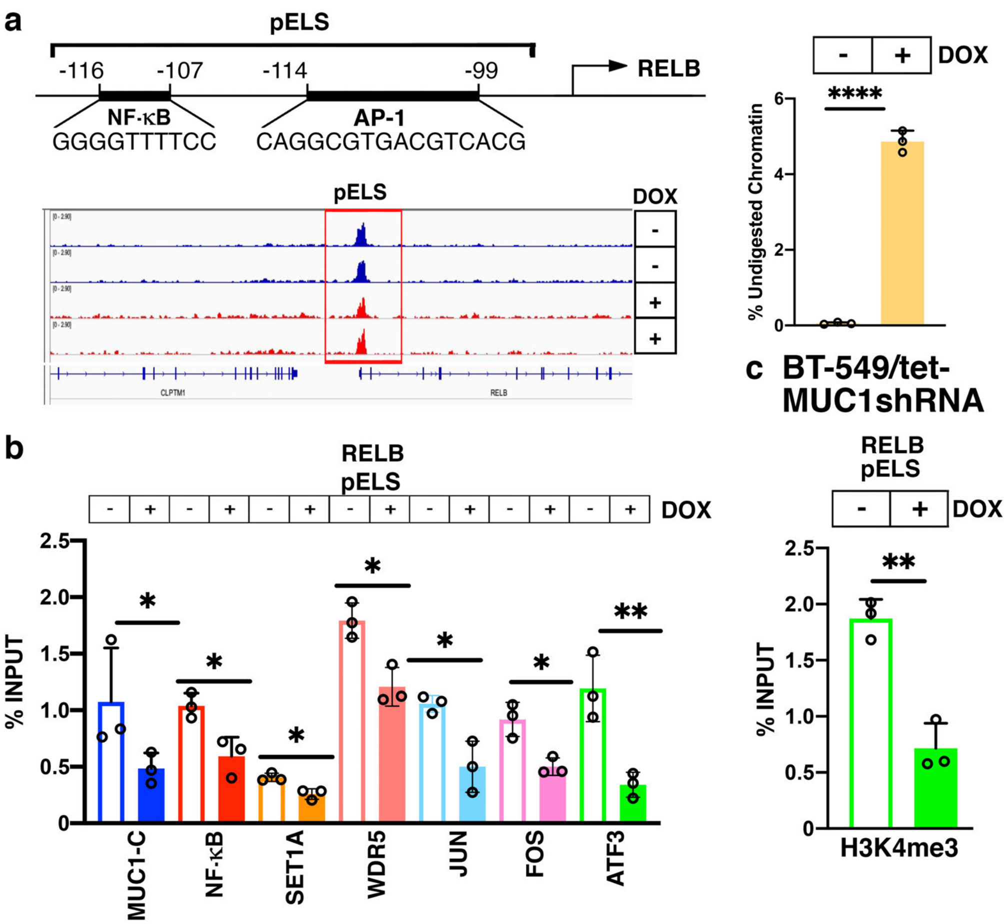

Supplemental Figure S7. MUC1-C and WDR5 regulate *RELB* activation. **a.** Genome browser snapshot of ATAC-seq data from the *RELB* pELS region in BT-549/tet-MUC1shRNA cells treated with vehicle or DOX for 7 days (left). Chromatin was analyzed for accessibility by nuclease digestion (right). The results are expressed as % undigested chromatin (mean±SD and individual values). **b and c.** Soluble chromatin from BT-549/tet-MUC1shRNA cells treated with vehicle or DOX for 7 days was precipitated with antibodies against the indicated proteins. The DNA samples were amplified by qPCR with primers for the *RELB* pELS region. The results (mean±SD of 3 determinations) are expressed as percent input.

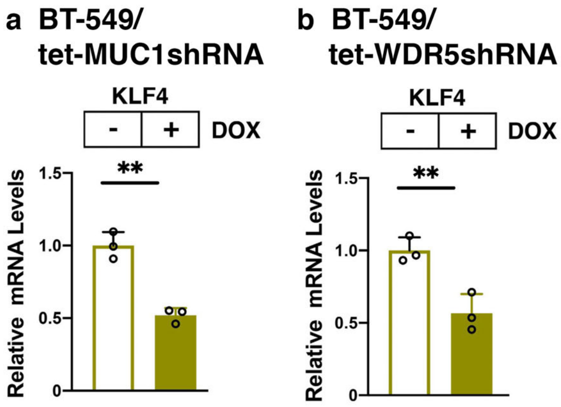

Supplemental Figure S8. Regulation of KLF4 and NOTCH1 expression. **a and b.** BT-549/tet-MUC1shRNA (**a**) and BT-549/tet-WDR5shRNA (**b**) cells treated with vehicle or DOX for 7 days were analyzed for KLF4 mRNA levels by qRT-PCR. The results (mean±SD of 3 determinations) are expressed as relative mRNA levels compared to that obtained for vehicle treated cells (assigned a value of 1).

Supplemental Figure S9. Uncut gels. Pages 19-37 display the uncut gels for the article figures. Pages 38-60 display the uncut gels for the supplementary figures.

Supplemental Table S1. Primers used for qRT-PCR analysis.

**NOTCH1**	**FWD**	GGGCTAACAAAGATATGCAG
	**REV**	ACTGAACCTGACCGTACAGTTGGCAAAGTGGTCCAG
**MUC1-C**	**FWD**	TACCGATCGTAGCCCCTATG
	**REV**	CTCACCAGCCCAAACAGG
**E2F1**	**FWD**	TATGGTGATCAAAGCCCCTC
	**REV**	AGATGATGGTGGTGGTGACA
**ACTIN**	**FWD**	GATGAGATTGGCATGGCTTT
	**REV**	CACCTTCACCGTTCCAGTTT
**LGR5**	**FWD**	TTCCAACCTCAGCGTCTTC
	**REV**	GTATGTCAGAGCGTTTCCCG
**HIF1A**	**FWD**	CATAAAGTCTGCAACATGGAAGGT
	**REV**	ATTTGATGGGTGAGGAATGGGTT
**TRAF1**	**FWD**	CATGAGAGGGGAGTATGATG
	**REV**	GAAGAAGAGTGGGCATCCAC
**RELB**	**FWD**	AGCCCGTCTATGACAAGAAATC
	**REV**	GCCCGCTTTCCTTGTTAATTC
**BATF3**	**FWD**	GATGACAGGAAGGTCCGAAG
	**REV**	CATGGTGTTTTCTTGCTCCAG
**JDP2**	**FWD**	GCTGAAATACGCTGACATCCG
	**REV**	TTTTCCTTCGCTCCTCTTCC
**WDR5**	**FWD**	AAAGCGTGAGGATATGGGATG

	**REV**	CAGATGCGACAGAGACCATC
**SETD1A**	**FWD**	GGCCAGATTCATCAACCACT
	**REV**	CGATCTTCTTCTGGGACTCG
**RBBP5**	**FWD**	GAACAAGCAATACCACAGCC
	**REV**	GCTCTCCATCTCTTCCACATG
**ASH2L**	**FWD**	ACCATTTTCTCCCCACTTCC
	**REV**	AGTACAGCACCGATTTCACAG
**c-JUN**	**FWD**	CCAAAGGATAGTGCGATGTTT
	**REV**	CTGTCCCTCTCCACTGCAAC
**JUNB**	**FWD**	CACCAAGTGCCGGAAGCGGA
	**REV**	AGGGGCAGGGGACGTTCAGA
**JUND**	**FWD**	CGCCTGGAAGAGAAAGTGAA
	**REV**	GTTGACGTGGCTGAGGACTT
**FOS**	**FWD**	CCAACCTGCTGAAGGAGAAG
	**REV**	AGATCAAGGGAAGCCACAGA
**ATF3**	**FWD**	CCTCTGCGCTGGAATCAGTC
	**REV**	TTCTTTCTCGTCGCCTCTTTTT
**KLF4**	**FWD**	GGCTGCGGCAAAACCTACAC
	**REV**	CGGGCGAATTTCCATCCAC
**BMI1**	**FWD**	GGTACTTCATTGATGCCACAAC
	**REV**	TGCTGGGCATCGTAAGTATC
**SALL4**	**FWD**	GCCGAAAGCATCAAGTCAAAG
	**REV**	GATAAACGTGGAAGGGAGACTG

**GAPDH**	**FWD**	CCATGGAGAAGGCTGGGG
	**REV**	CAAAGTTGTCATGGATGACC
**SETD1B**	**FWD**	CCTCAACCTCATCCACATCAG
	**REV**	GTCATCGTTCTCAGACTCGTC

Supplemental Table S2. Primers used for ChIP-PCR.

**ATF3**	**FWD**	AGCTGTGCCATAGAAACCTC
	**REV**	CTCAACTGTTTCCCCTTCCTC
**TRAF1**	**FWD**	GGTGGAGTGTGAAGGACG
	**REV**	GACTCAGTTTCCCCTCACAG
**RELB**	**FWD**	TTGTCTCGTCCAGAGCAATG
	**REV**	GGGAACGGGAAAACCCC
**SETD1A**	**FWD**	TTGGAAACTGGAGTGATGGG
	**REV**	ACCTTCCTGATCCATCTTTGC
**SETD1B**	**FWD**	TGTTCTCCATGCCGTTAACC
	**REV**	GATTGGATTCTTTCGCGTGTG
**WDR5**	**FWD**	GCGTGCTGTCTGTGGAAA
	**REV**	GCCCTTCTAATGCACTTCCTAAA
**NOTCH1**	**FWD**	TCGGTCCTCCCTGATCC
	**REV**	GAGGCCAGCATGGAGAG
**NOTCH1**	**FWD**	CCTGGGACTACTTCTCGTTTG
	**REV**	GCAAATTTCAGTCGCCAGTTG
**RBBP5**	**FWD**	GTAGGTGAGGTAGAAATTCCAGG
	**REV**	CTTCCCTGGTTTCTTGCATTAG
**FOS**	**FWD**	TTGAGCCCGTGACGTTTAC
	**REV**	GGCTCAGTCTTGGCTTCTC
**KLF4**	**FWD**	TCCTTCGCTACAGCCTTTTC
	**REV**	CATAGCAACGATGGAAGGGAG

Uncut gels related to Figures 1-8

Figure 1 a

H3K4me3

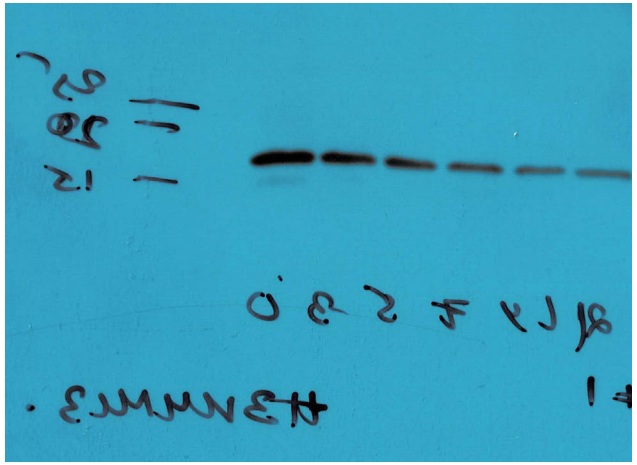

H3K4me1

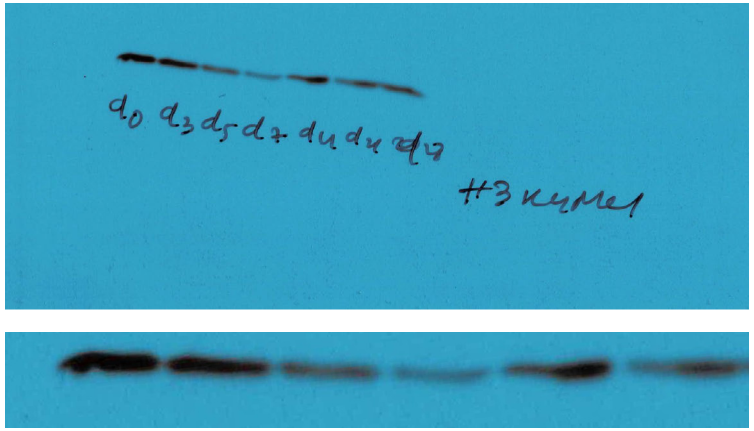

Total H3

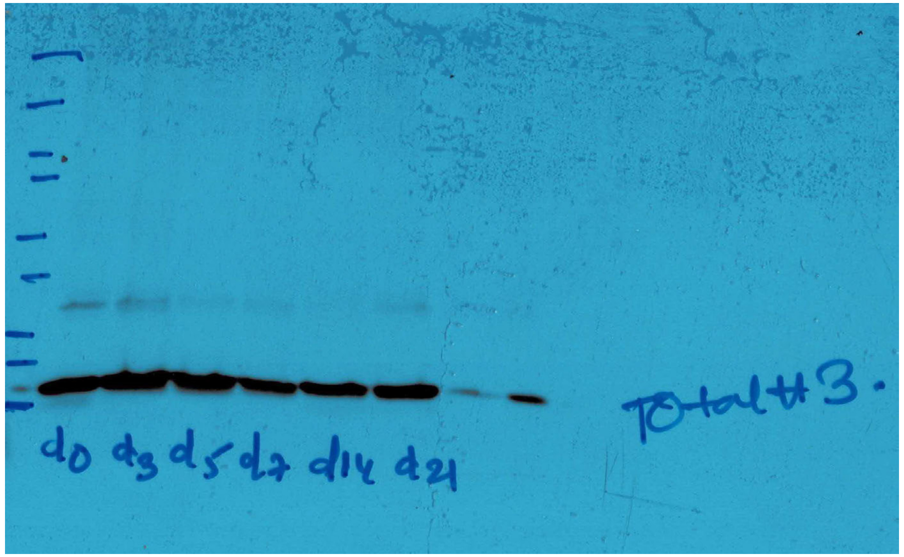

MUC1-C

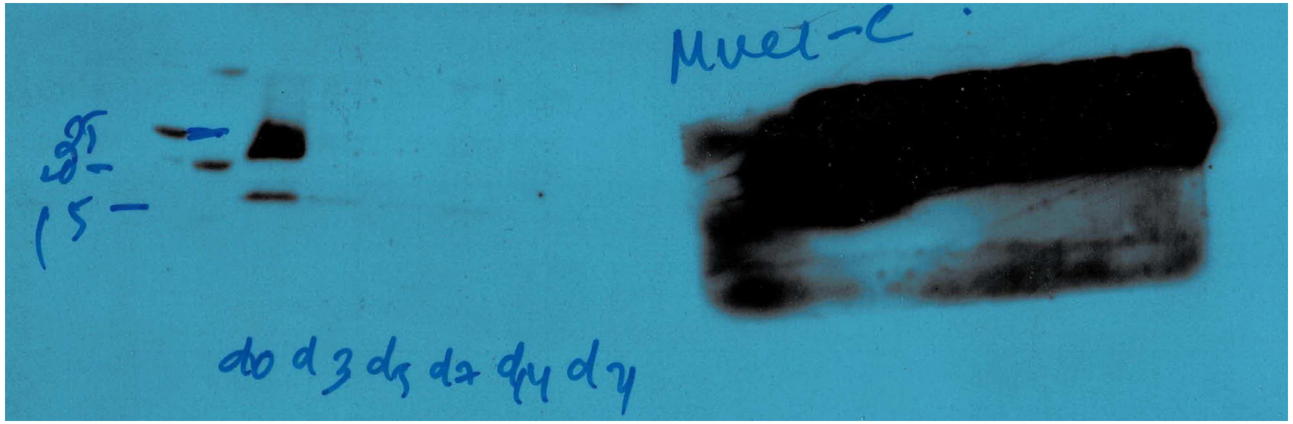

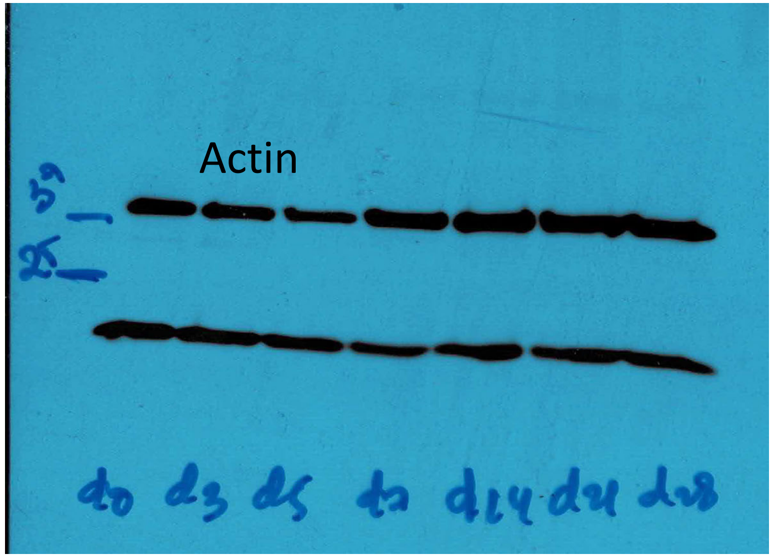

Figure 1 b

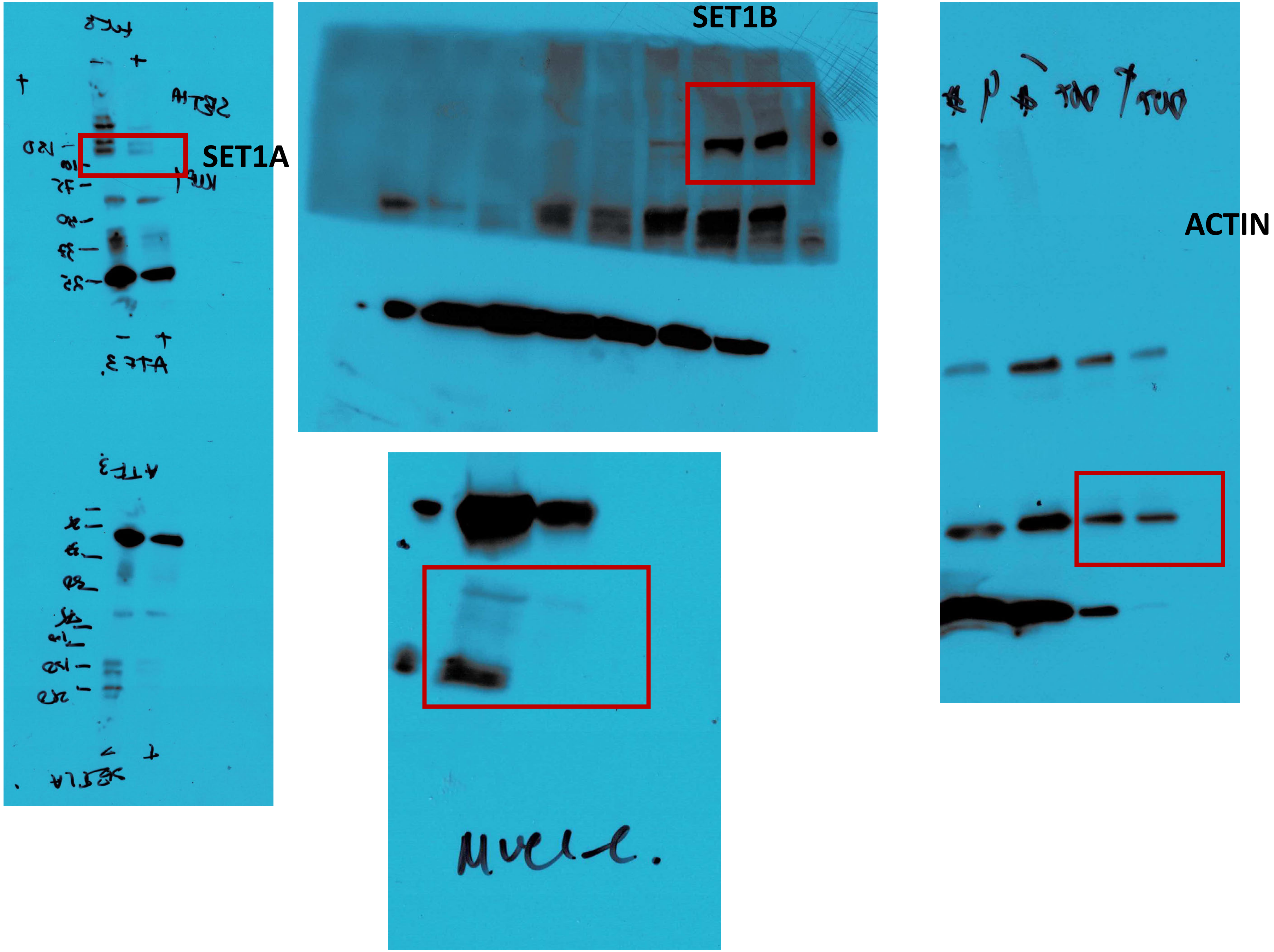

Figure 1 c

MDA-MB 468

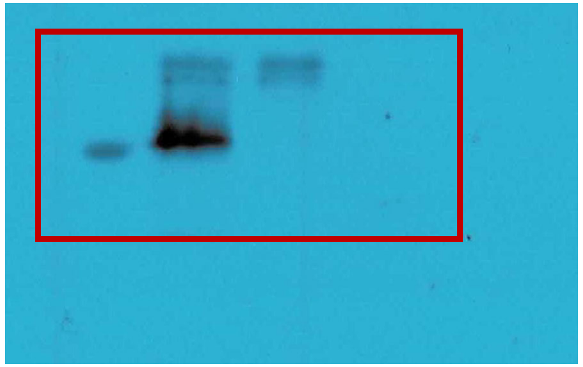

MUC1-C

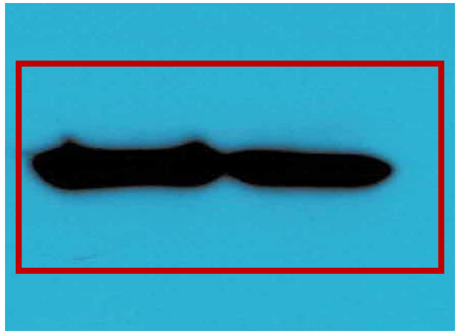

ACTIN

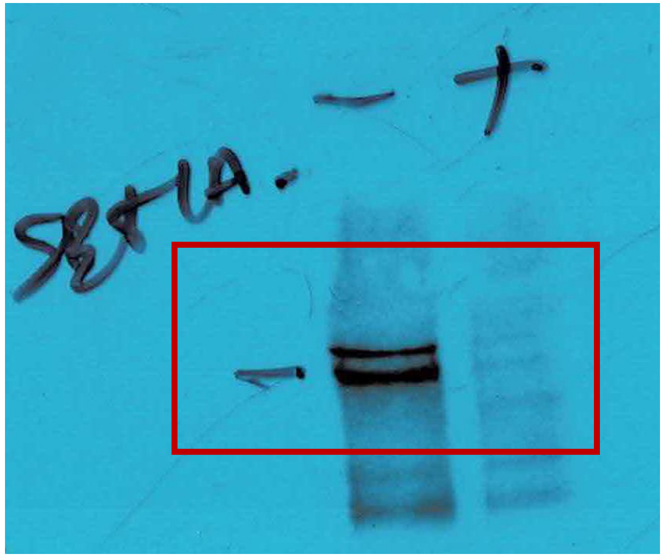

SET1A

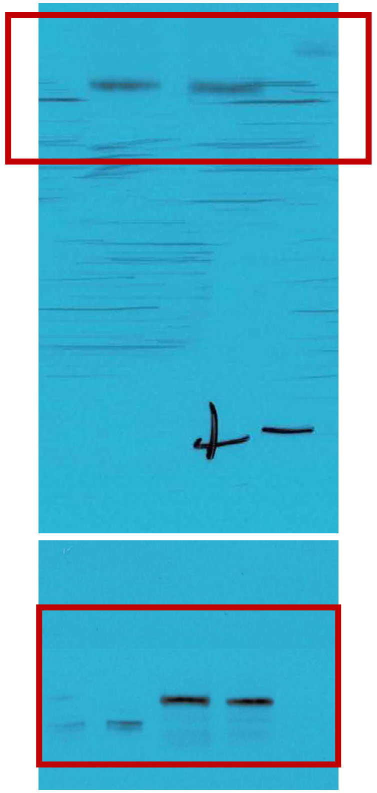

SET1B

Figure 1 d left

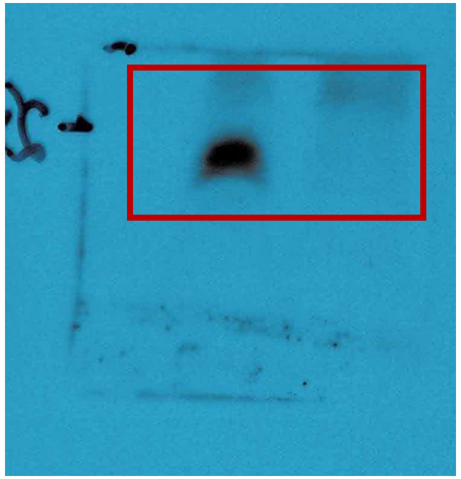

MUC1-C

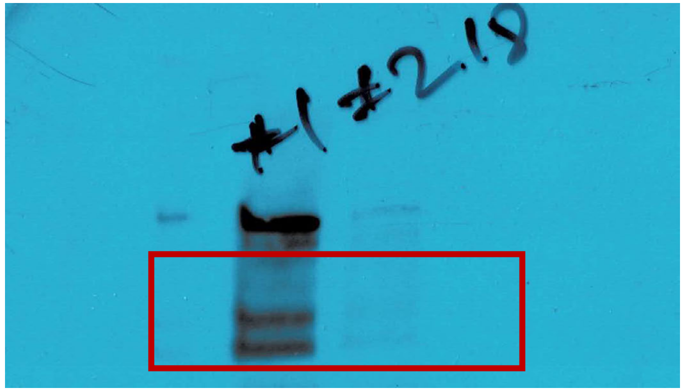

SET1A

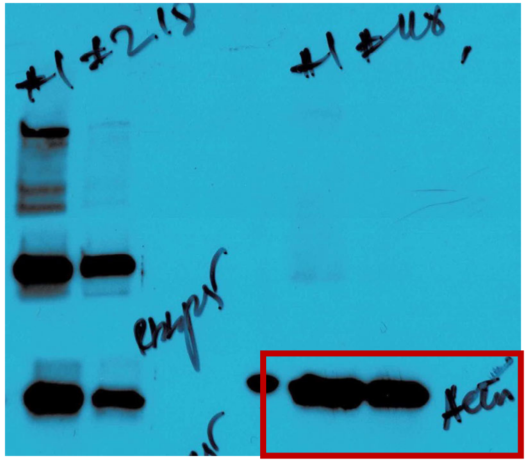

ACTIN

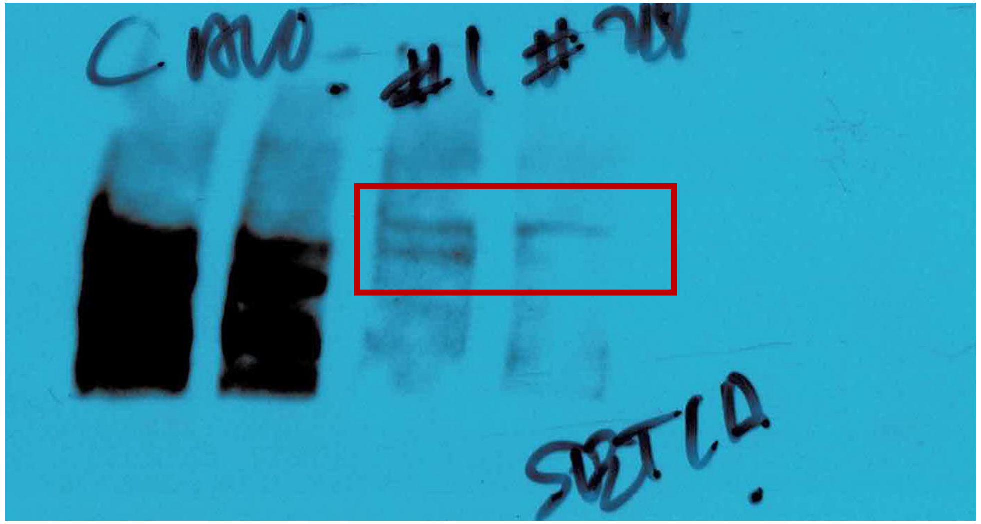

Figure 1 e

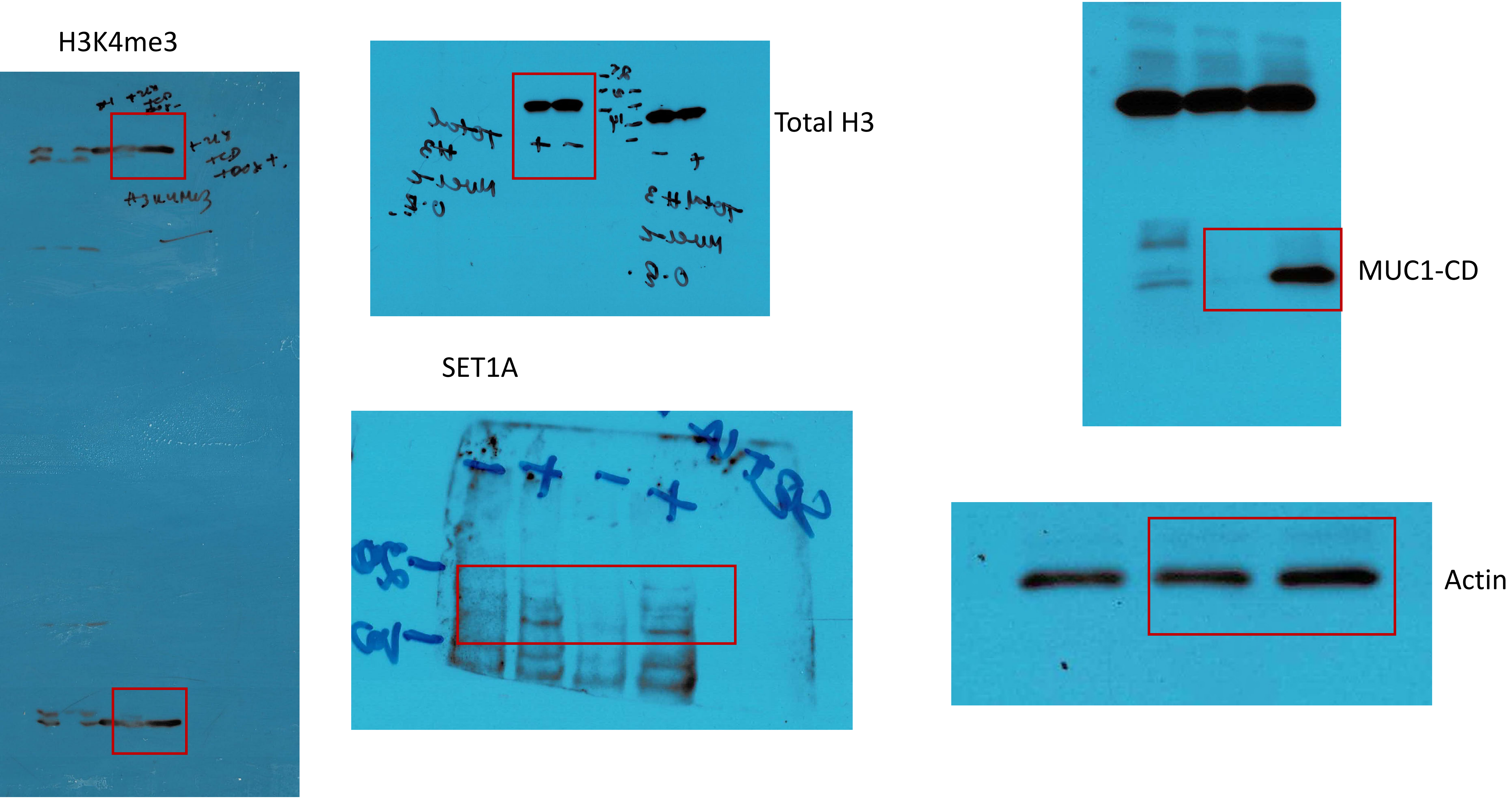

Figure 1 f

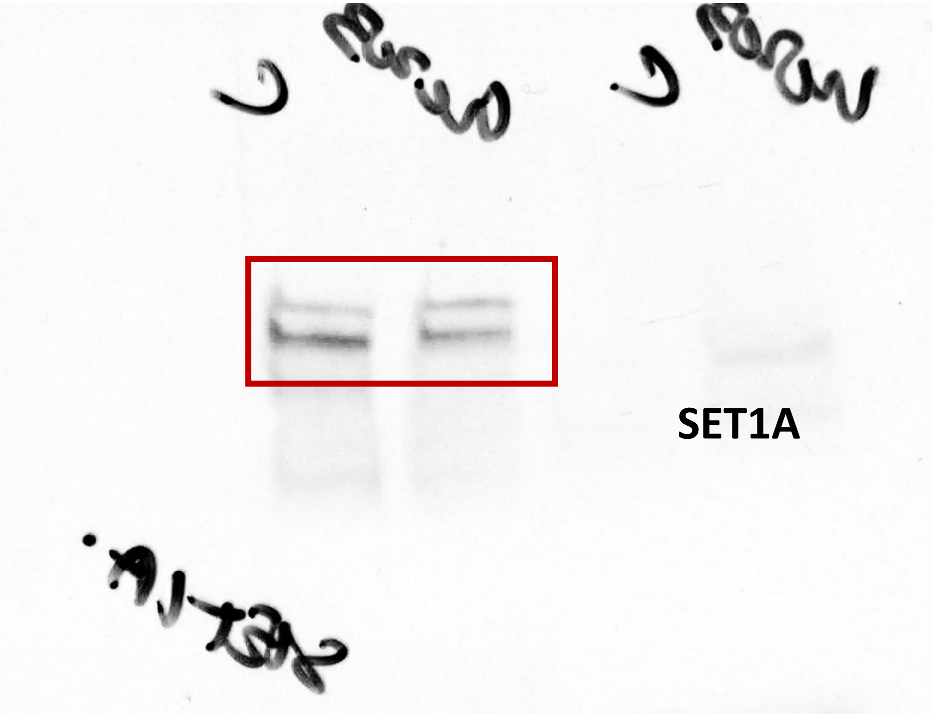

SET1A

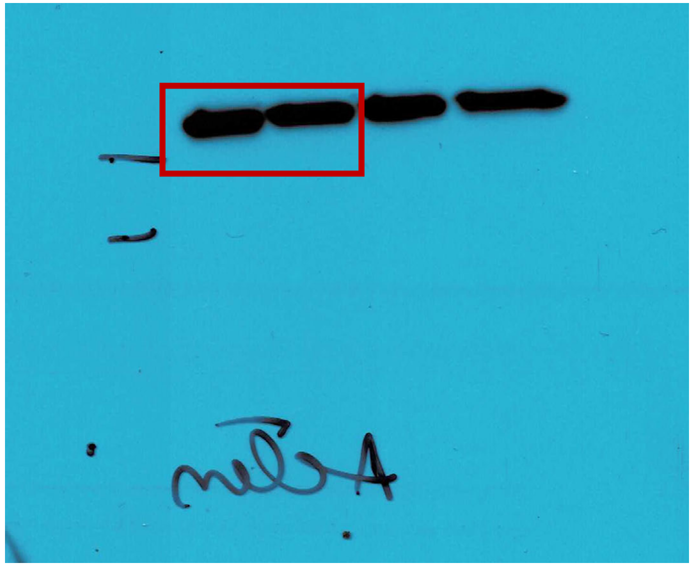

Actin

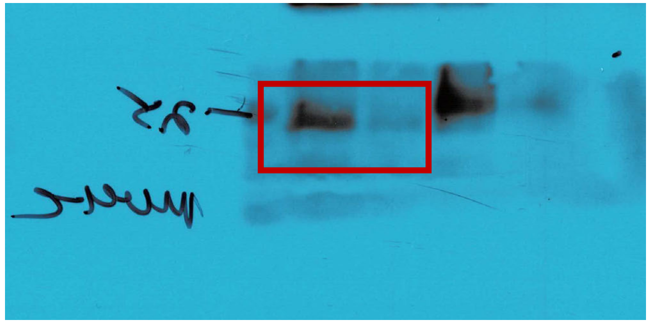

MUC1-C

Figure 2 a

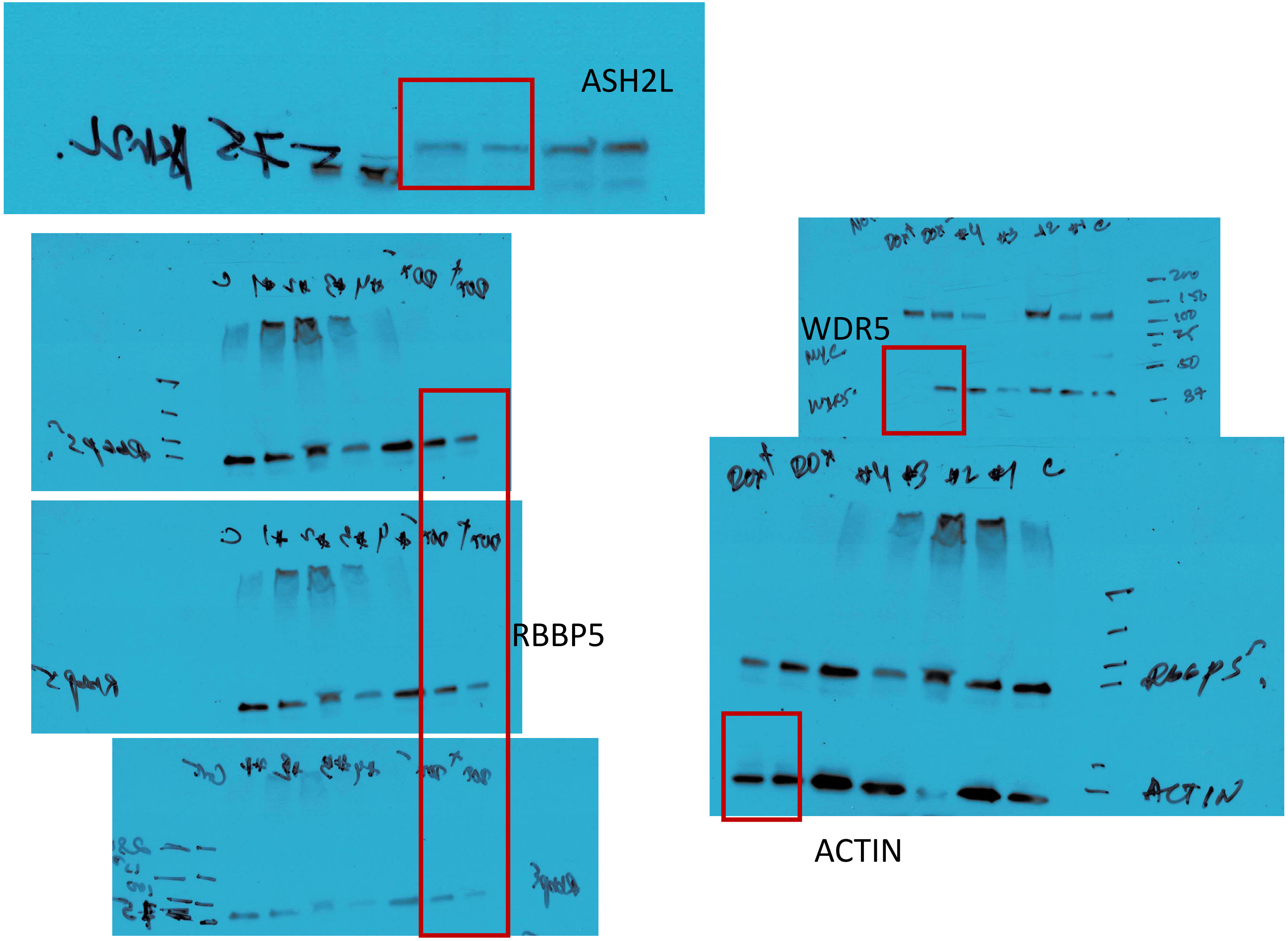

Figure 2 b

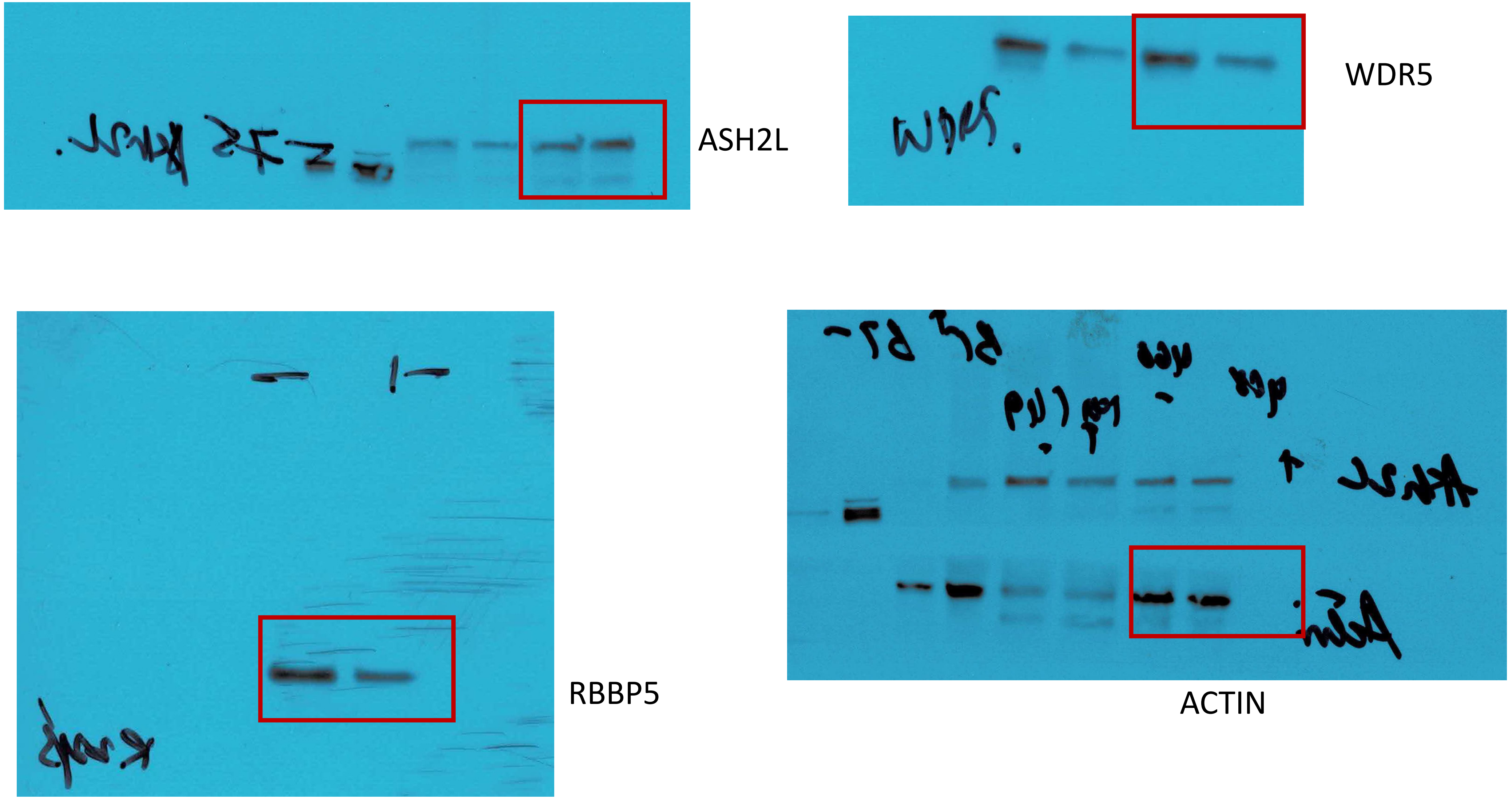

Figure 2 c

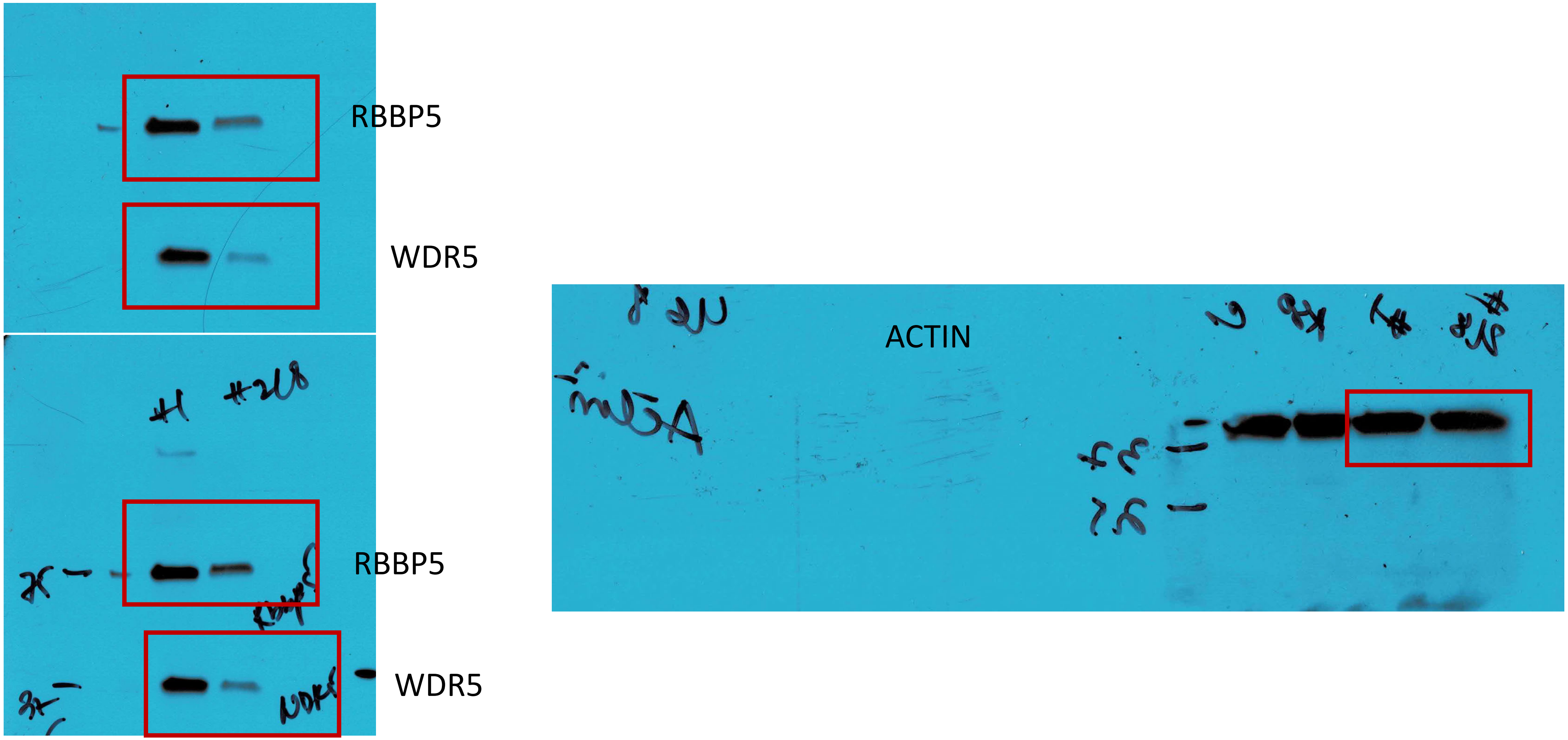

Figure 2 d

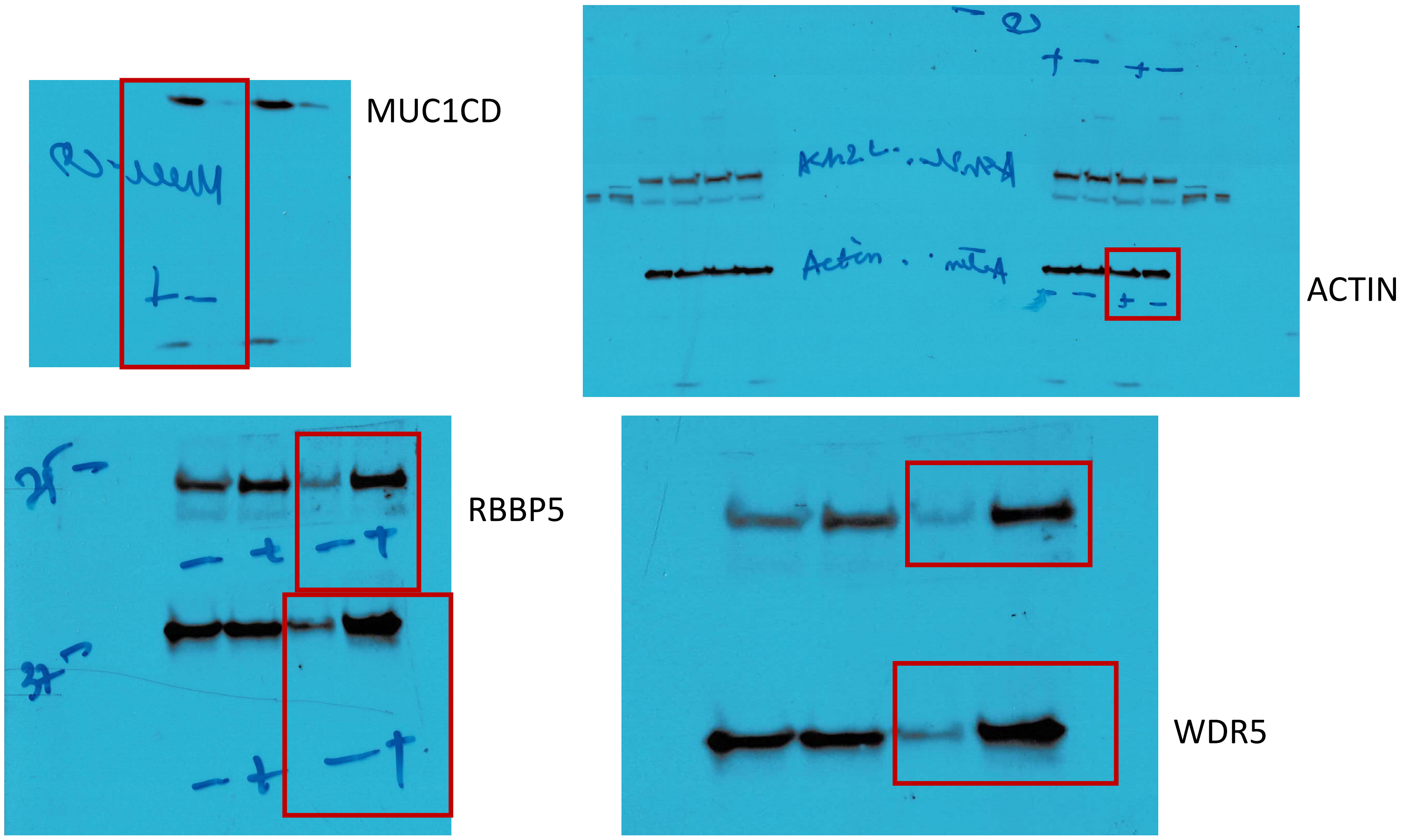

Figure 2 e

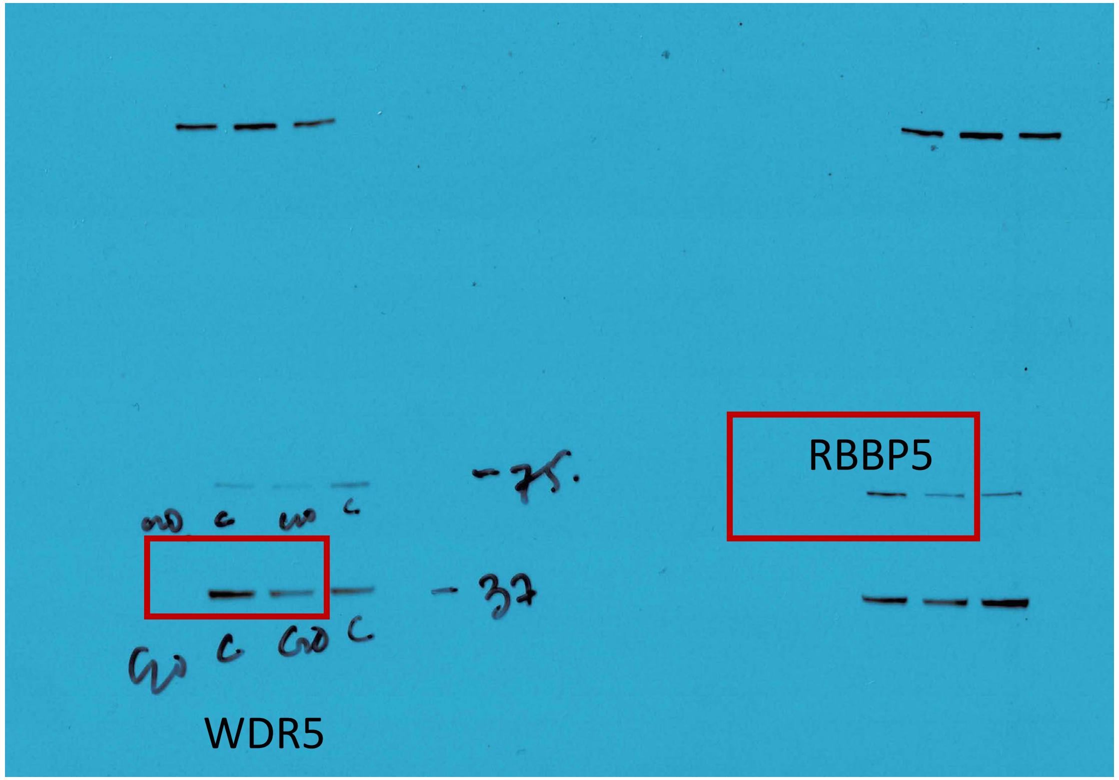

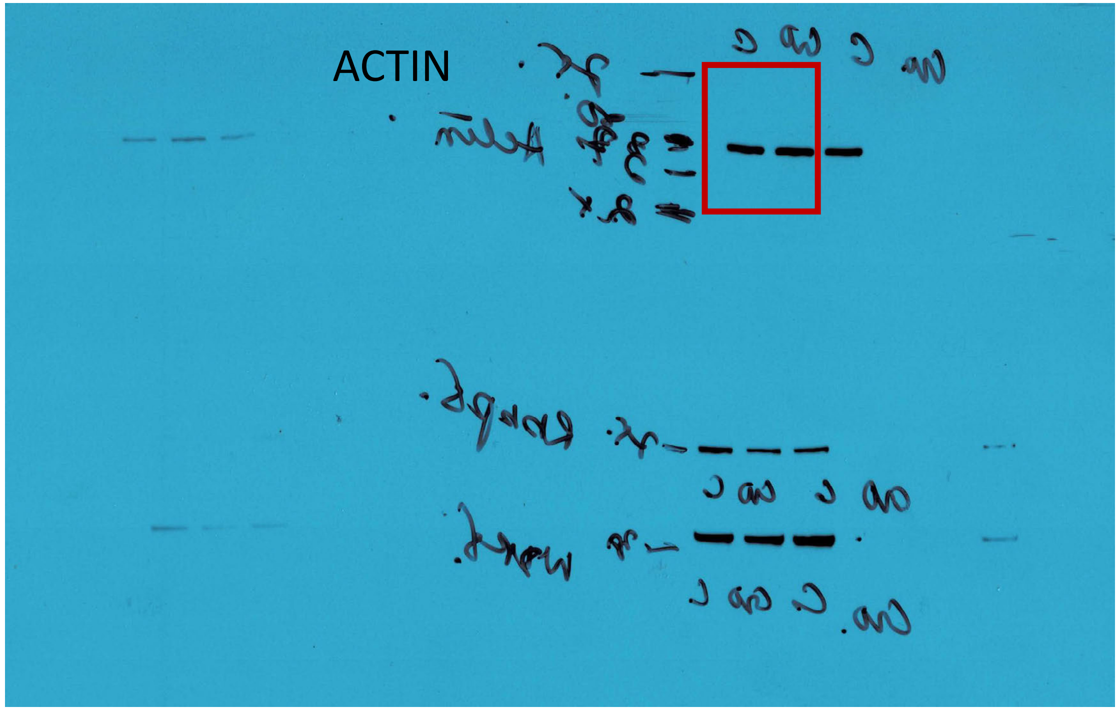

Figure 2 f

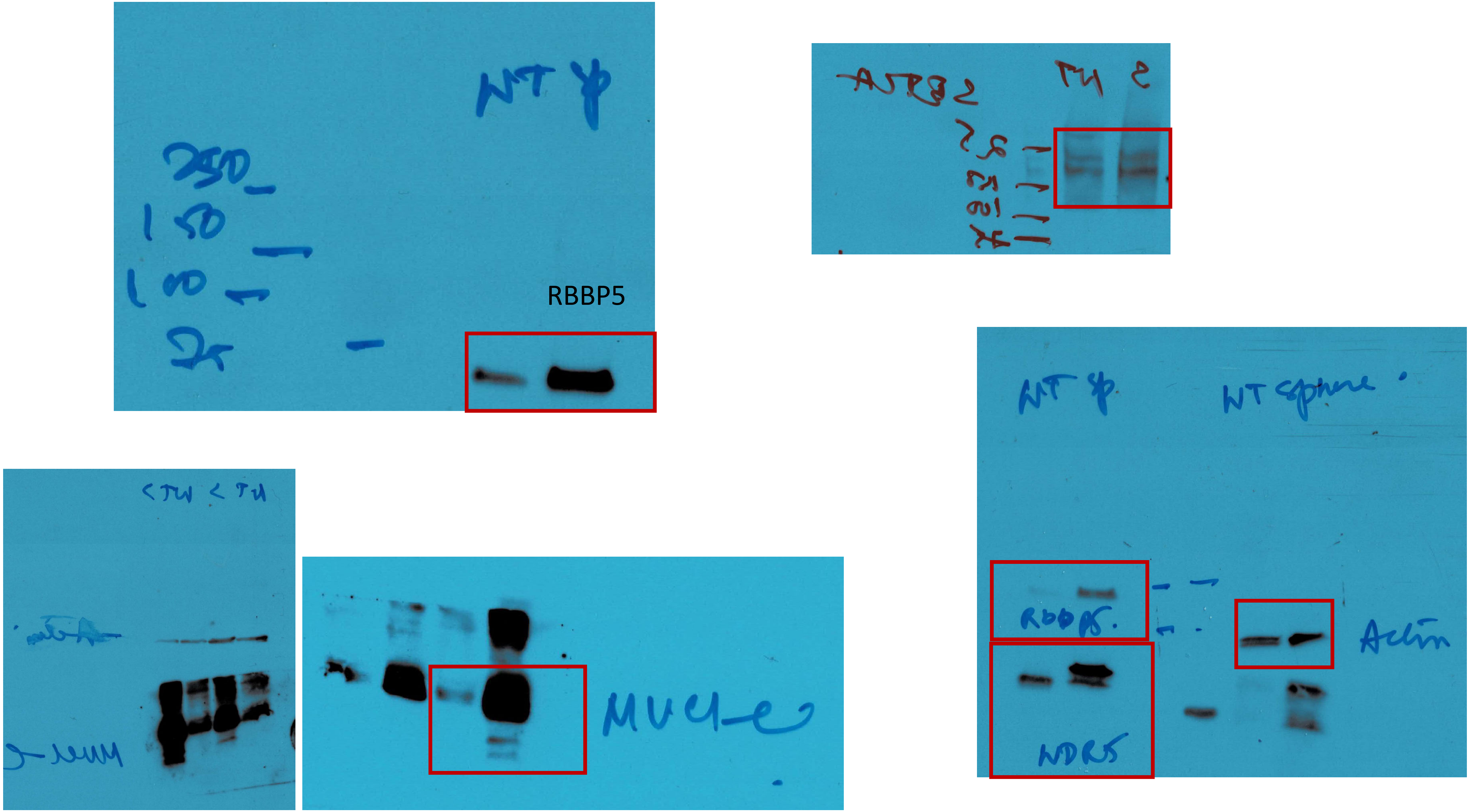

Figure 2 g

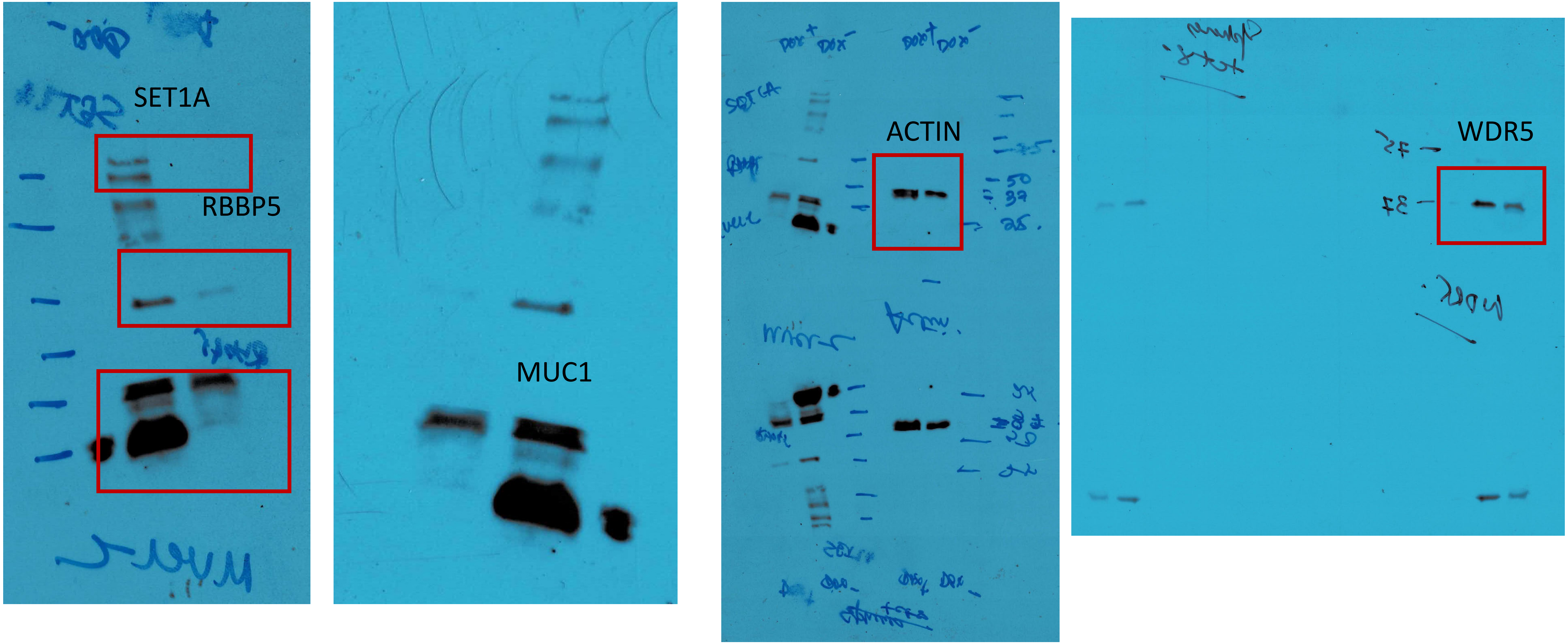

Figure 3 a

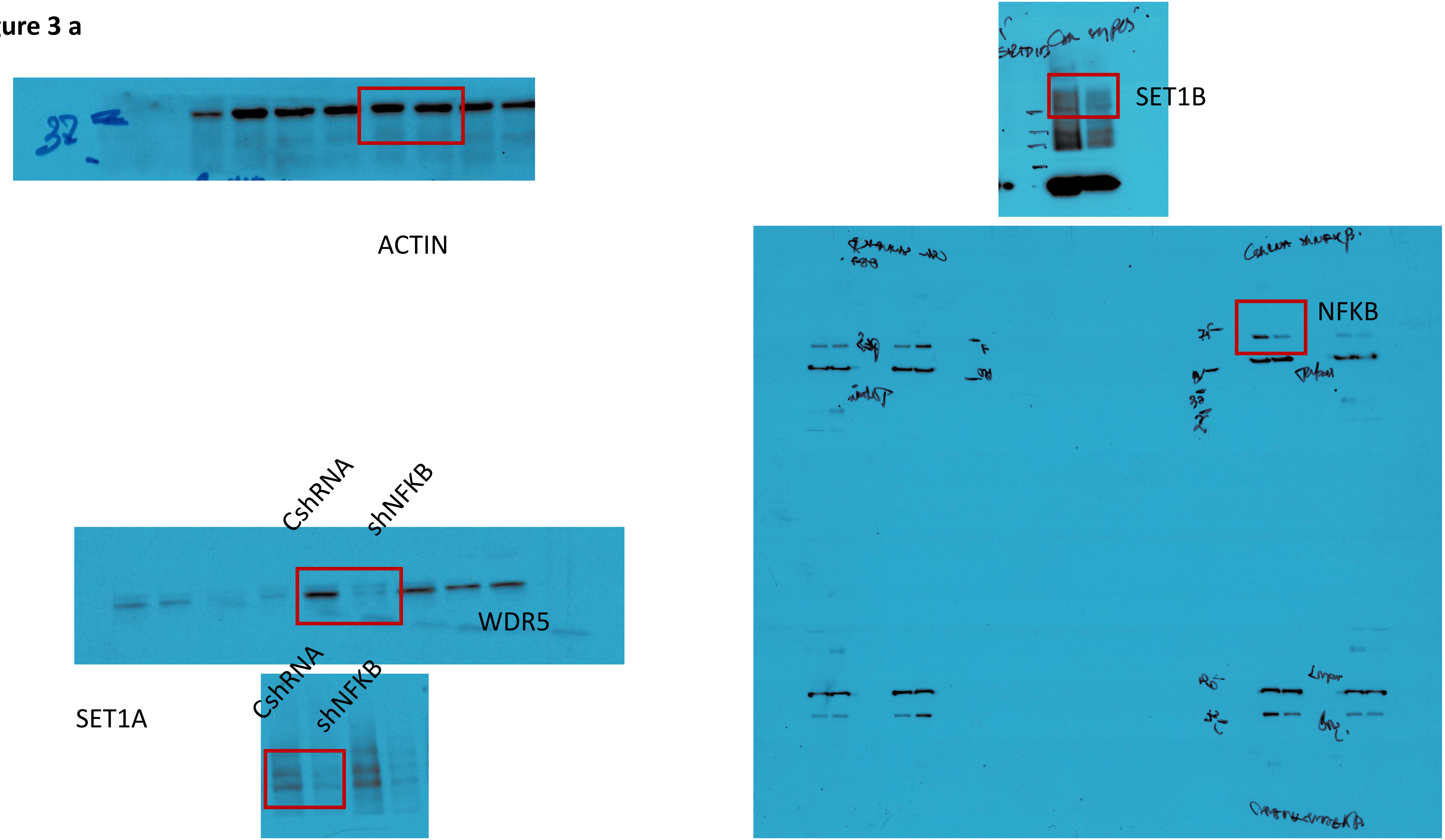

Figure 3 b

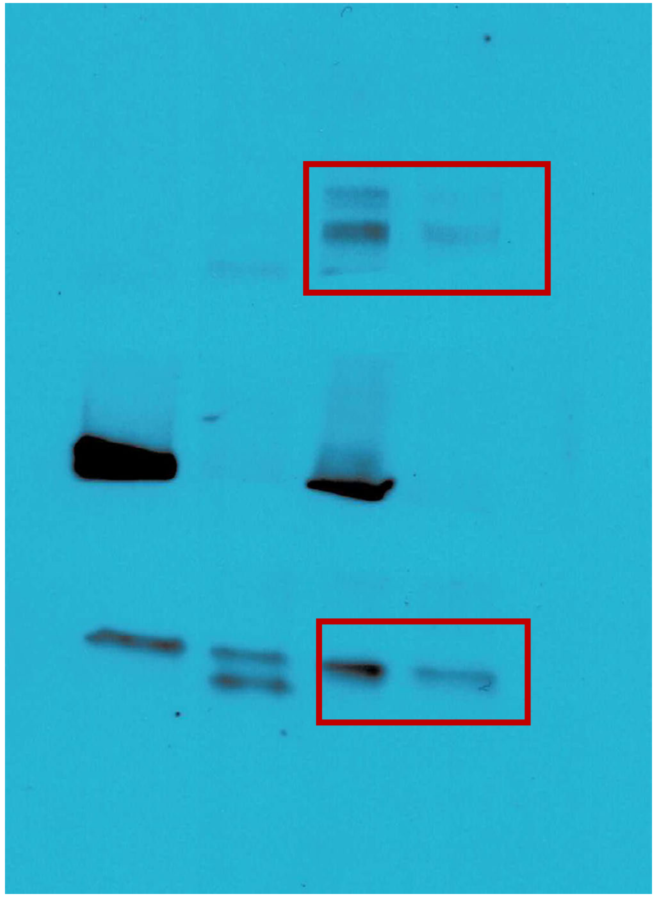

SET1A

WDR5

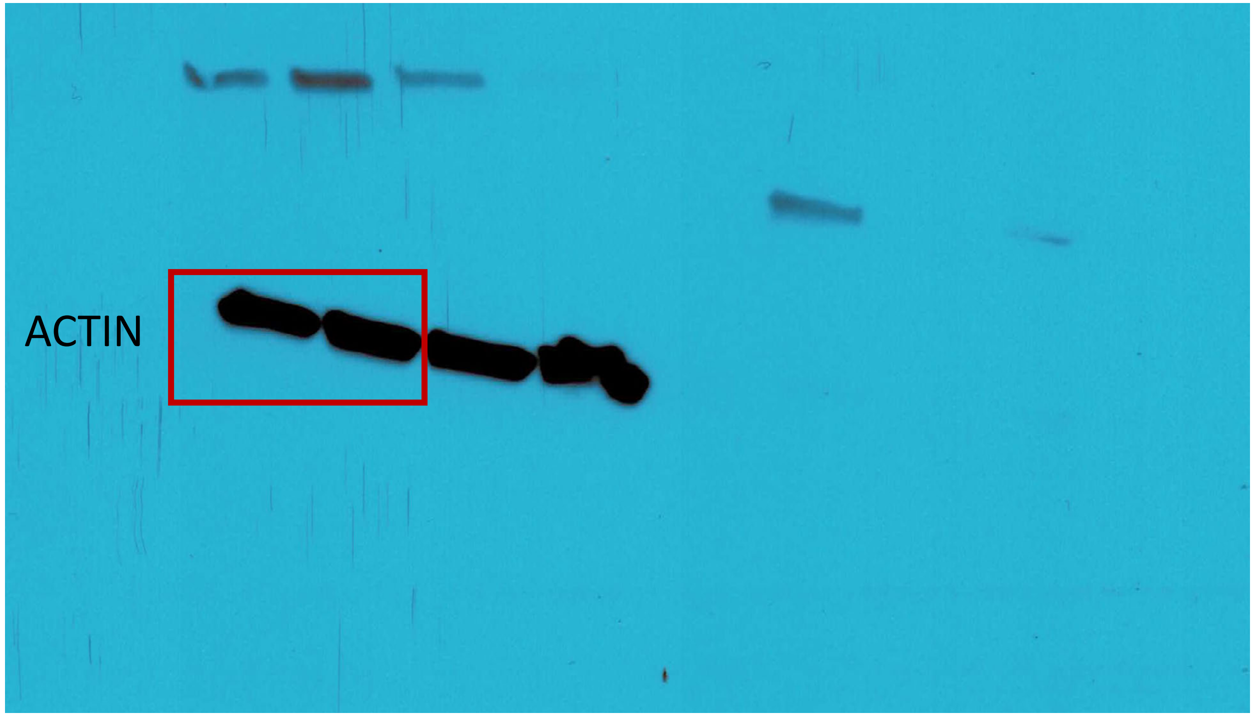

ACTIN

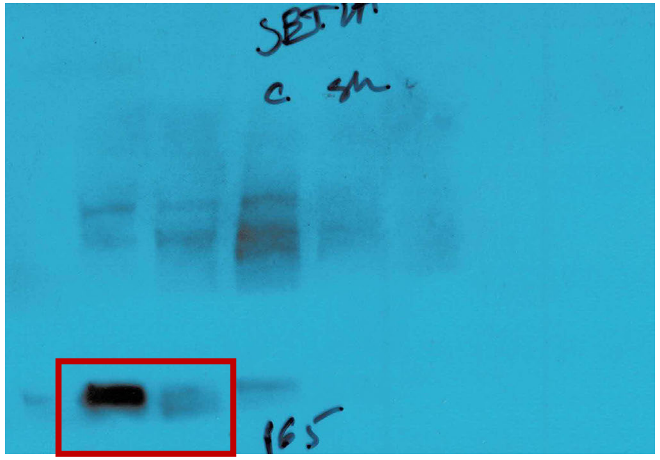

NFKB

Figure 3 c

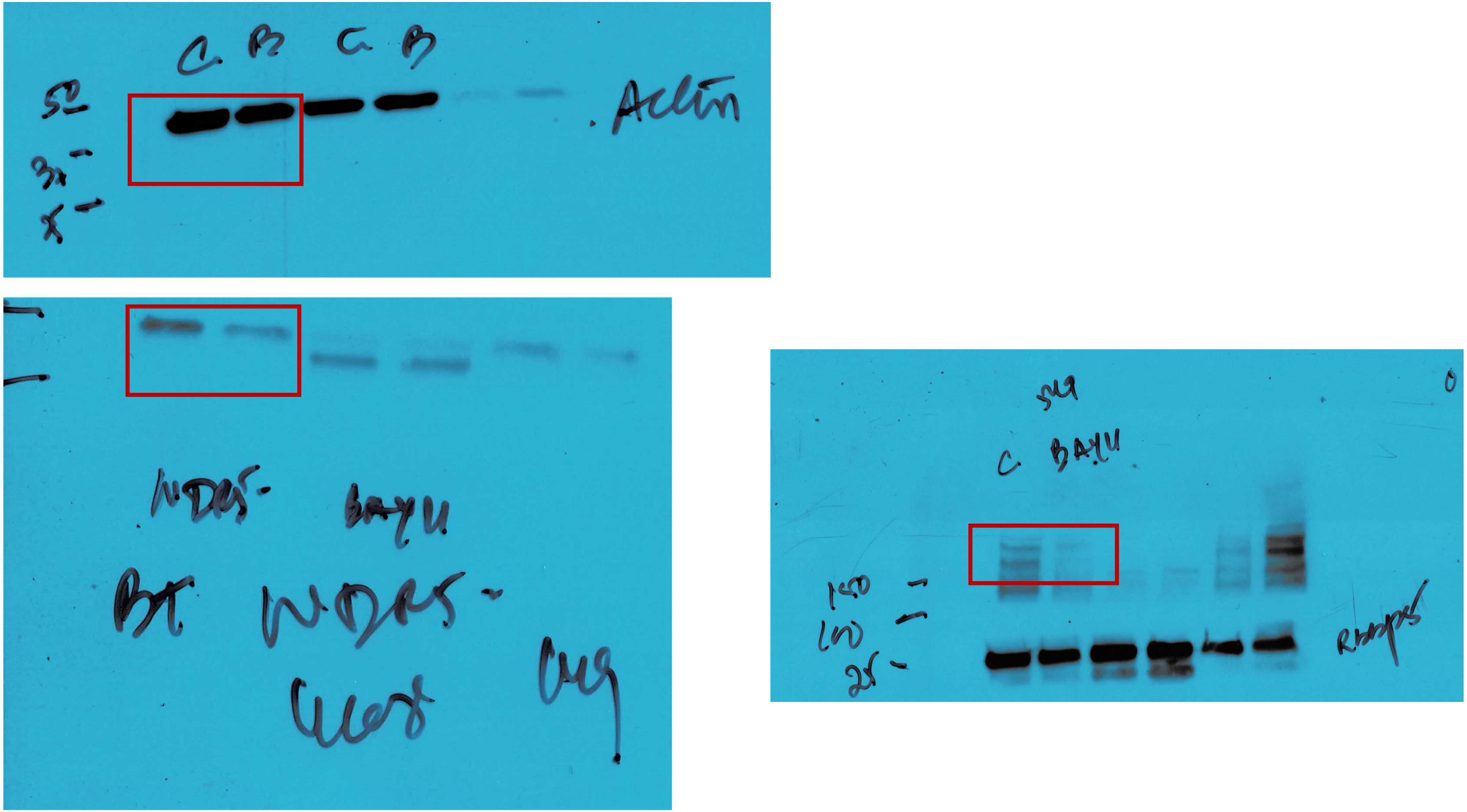

Figure 4 a

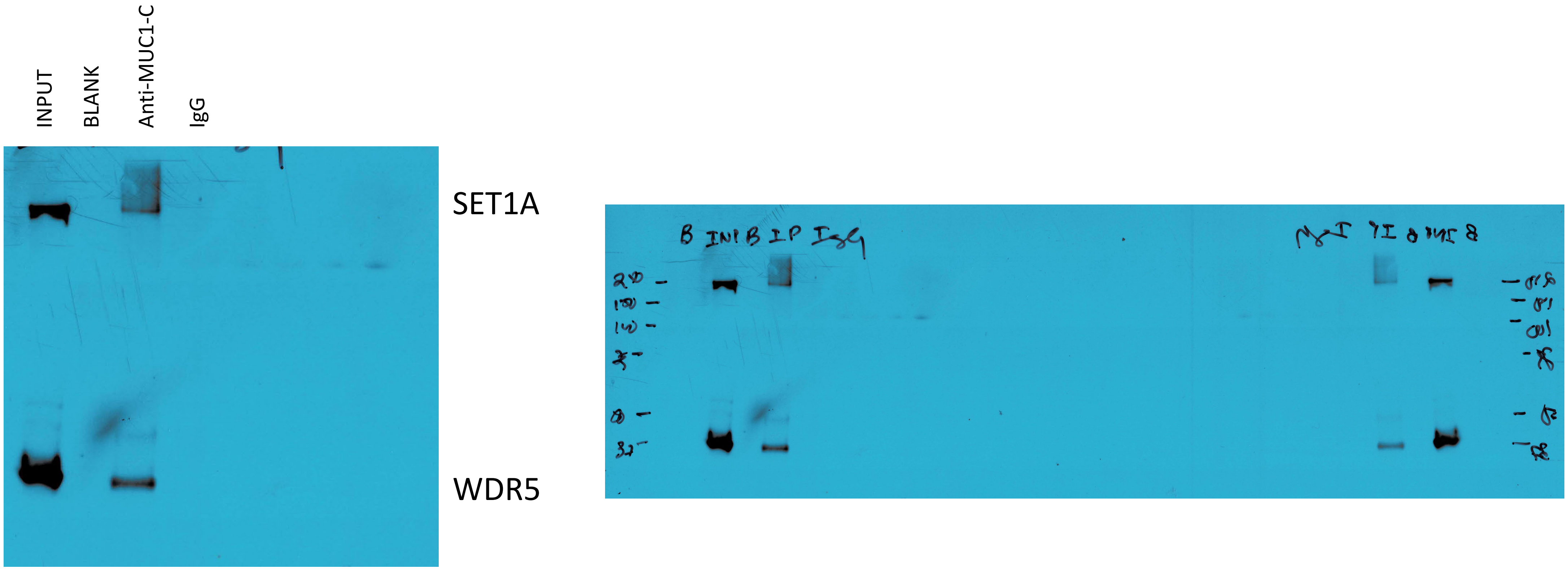

Figure 4 e

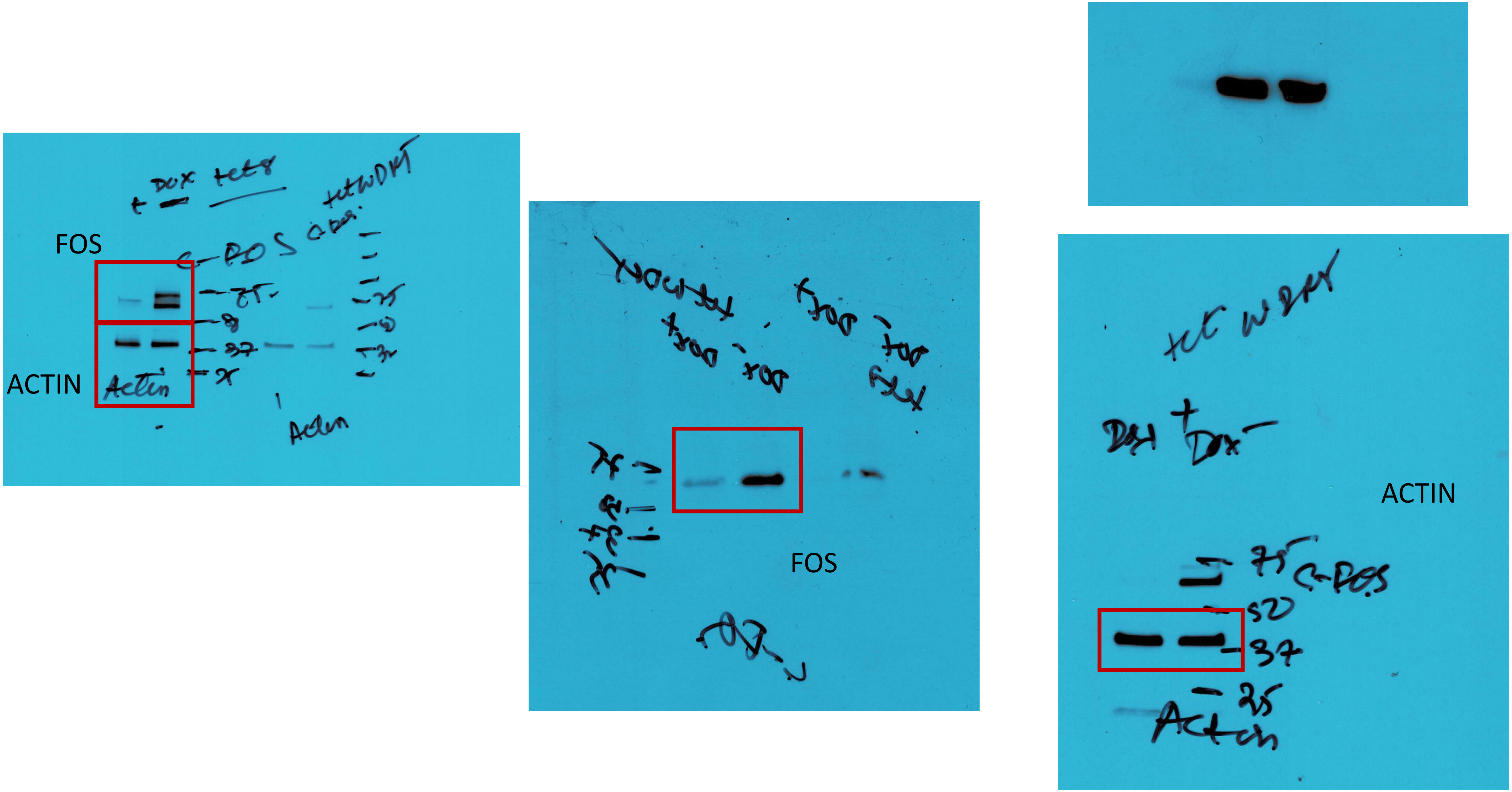

Uncut gels related to Supplementary Figures S1-S8

Fig. S1a

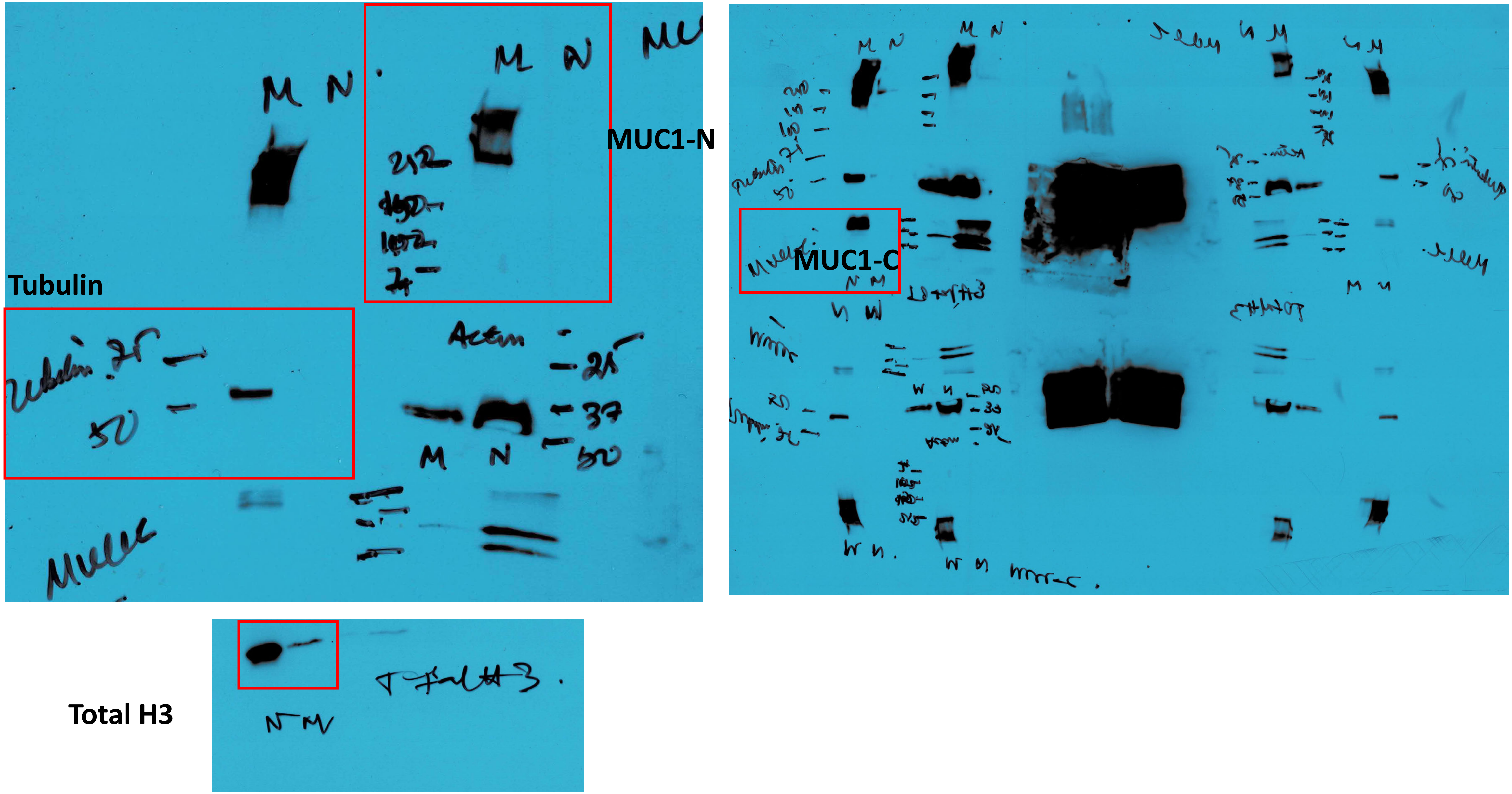

Fig. S1b

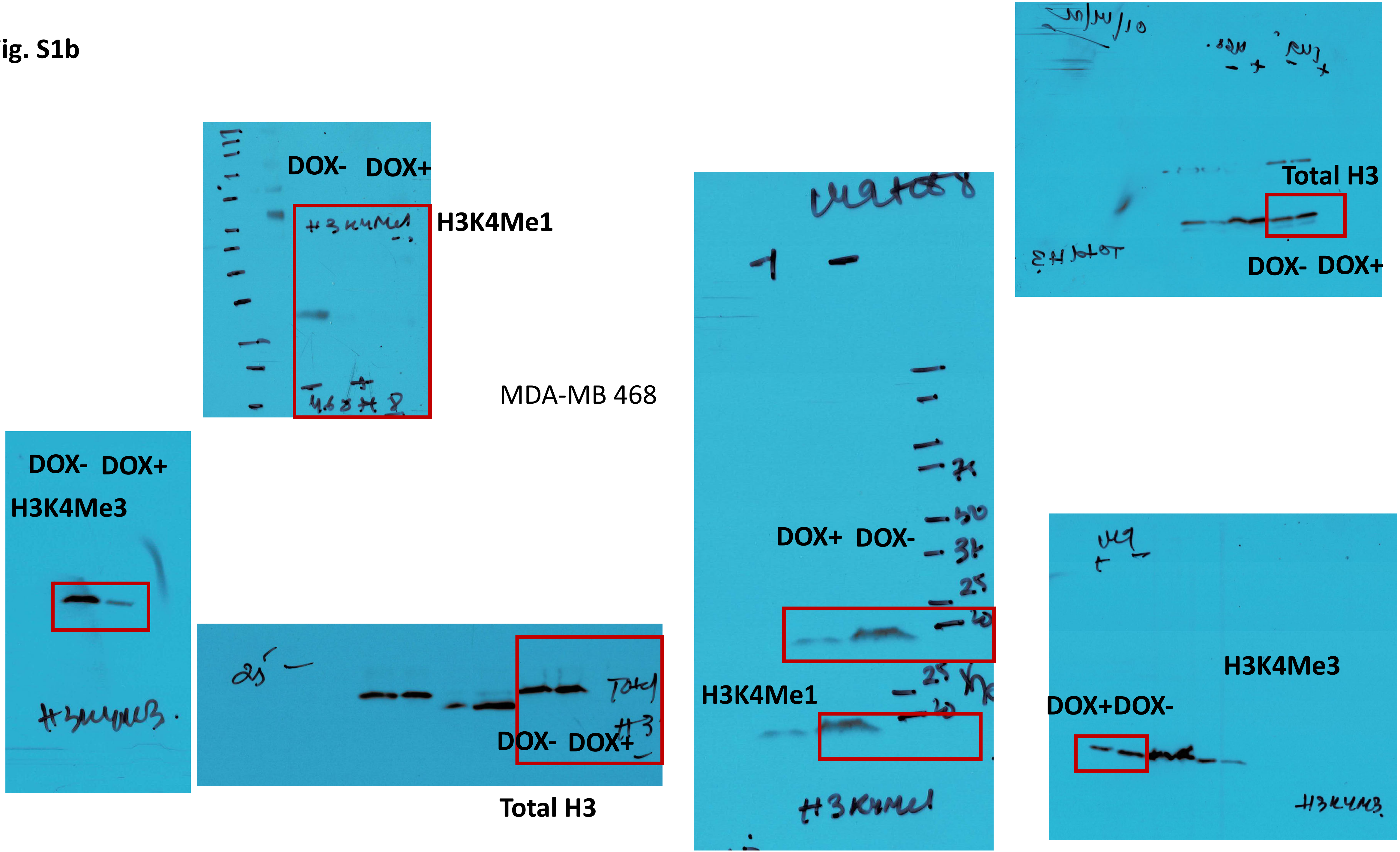
DOX- DOX+
H3K4Me1
MDA-MB 468
DOX- DOX+
H3K4Me3
DOX- DOX+
Total H3
DOX+ DOX-
H3K4Me1
Total H3
DOX- DOX+
H3K4Me3
DOX+DOX-

Fig. S1d

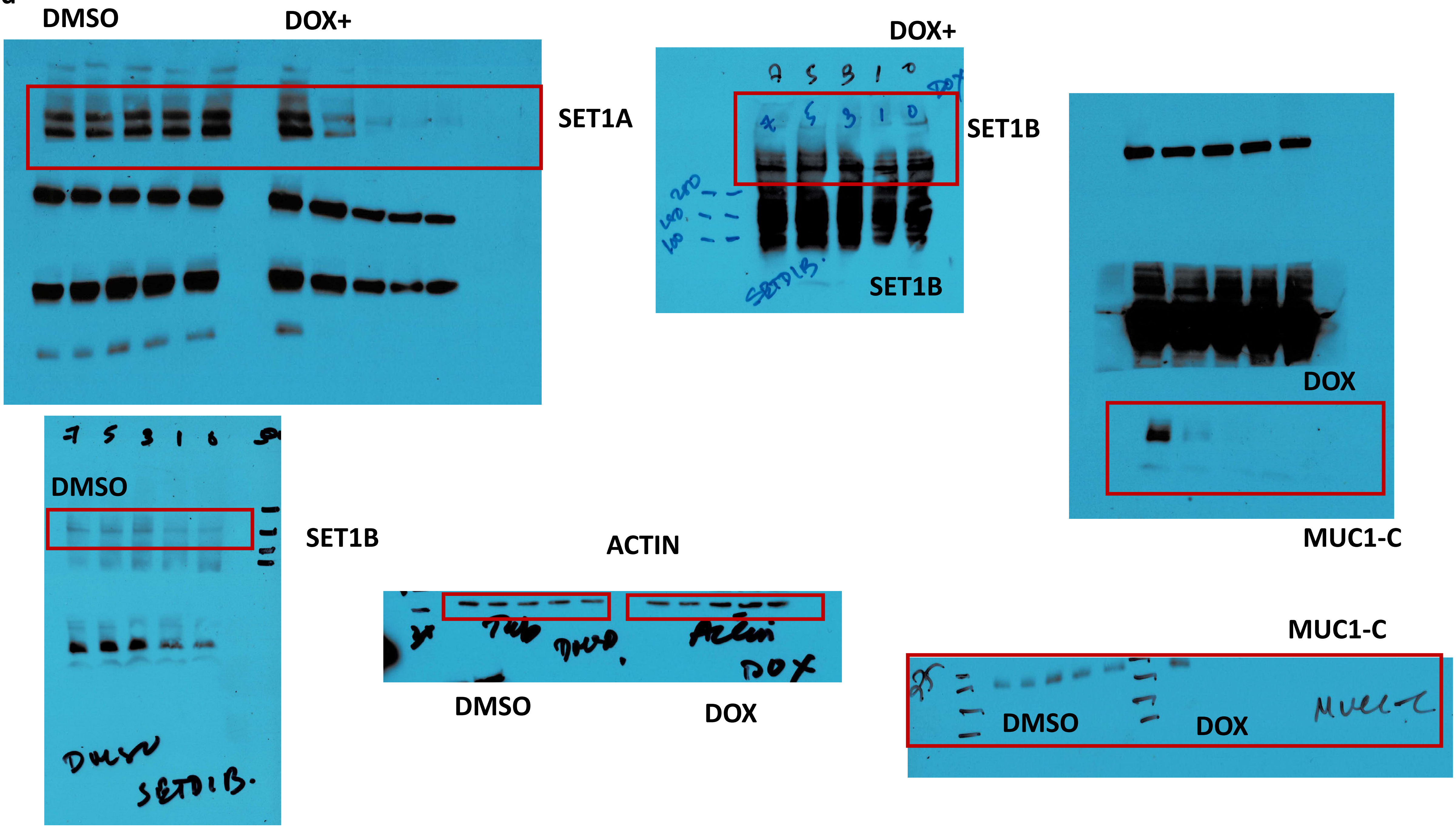
DMSO
DOX+
SET1A
DOX+
SET1B
SET1B
DOX
MUC1-C
DMSO
SET1B
ACTIN
DMSO
DOX
MUC1-C
DMSO
DOX

Fig. S1e

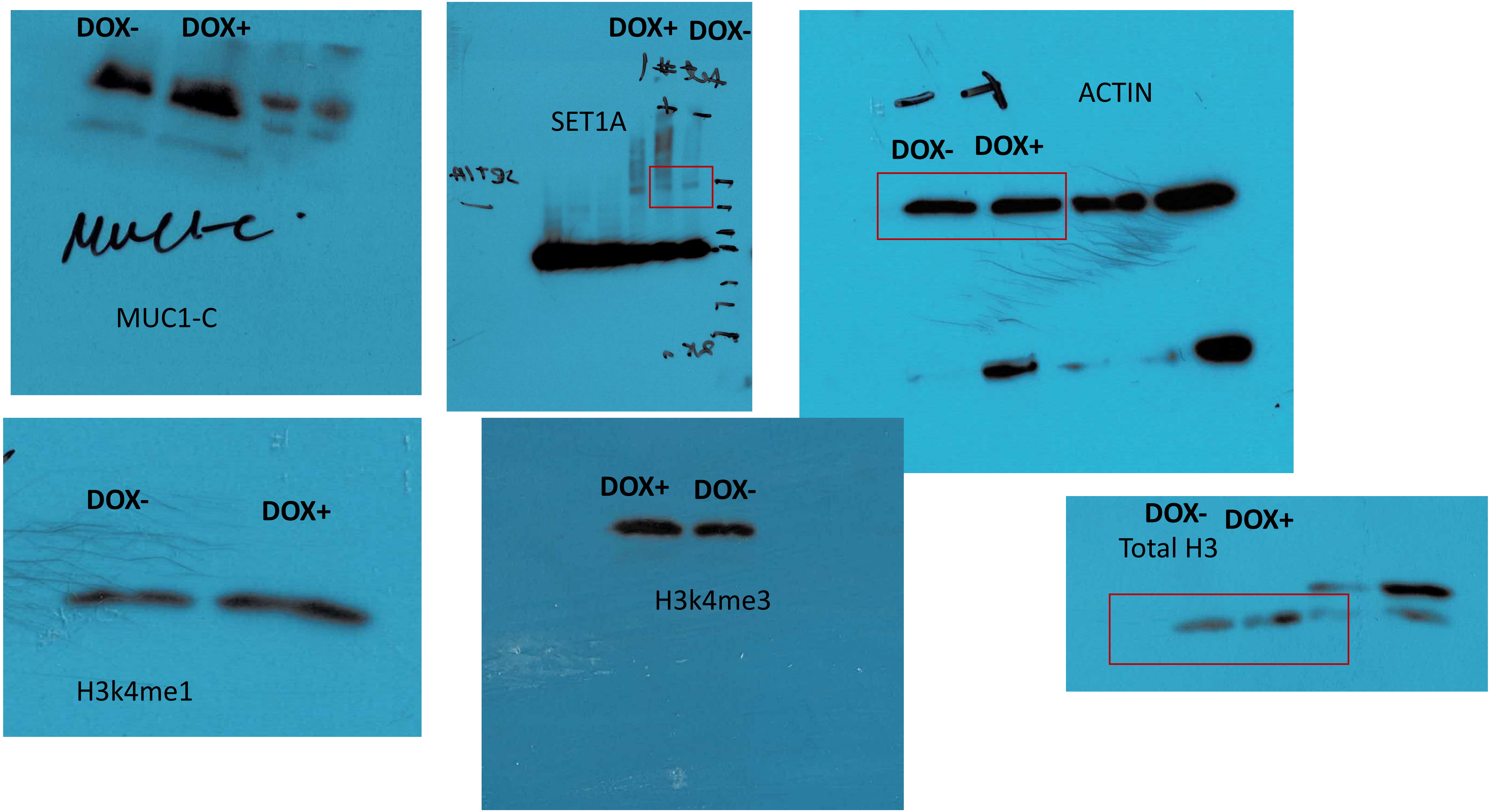
DOX-
DOX+
MUC1-C
DOX+
DOX-
SET1A
ACTIN
DOX-
DOX+
DOX-
DOX+
H3k4me1
DOX+
DOX-
H3k4me3
DOX-
DOX+
Total H3

Fig. S1f

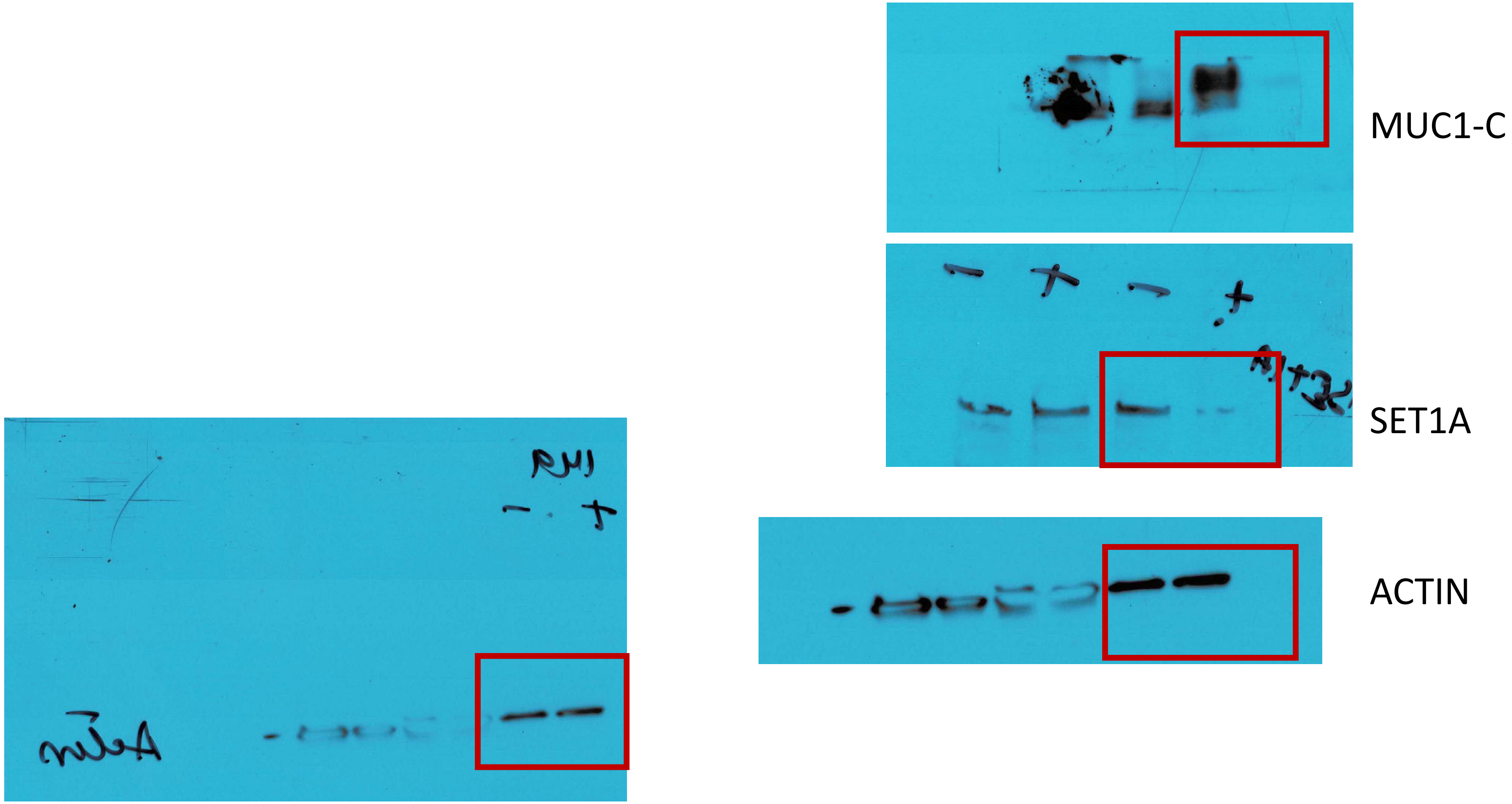

Fig. S1h

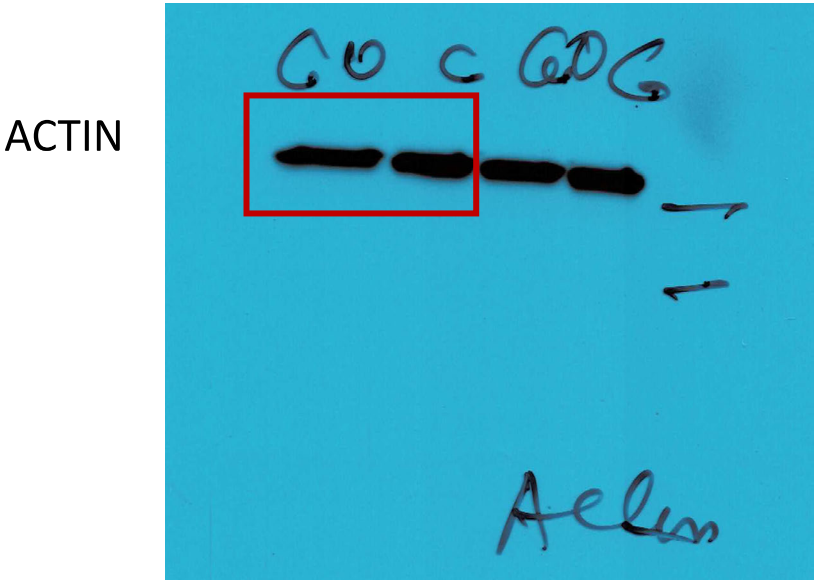

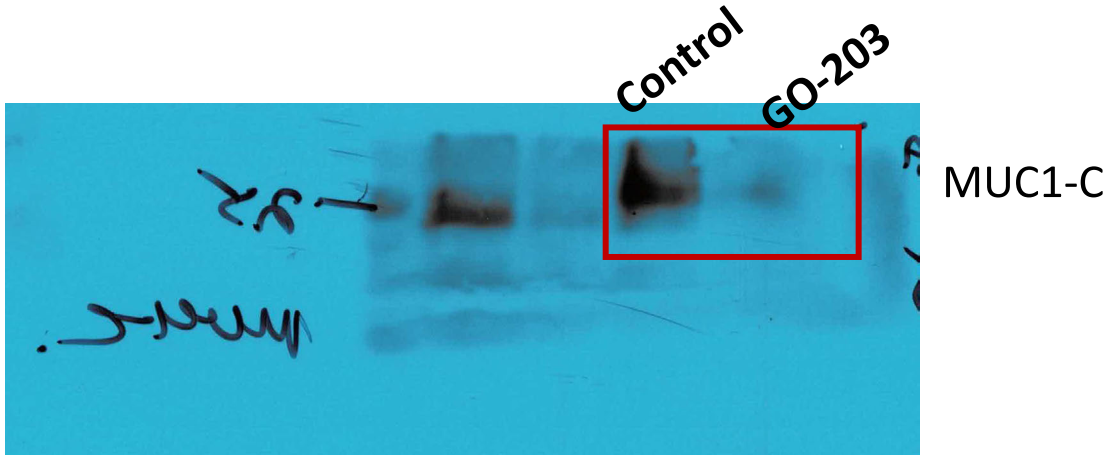

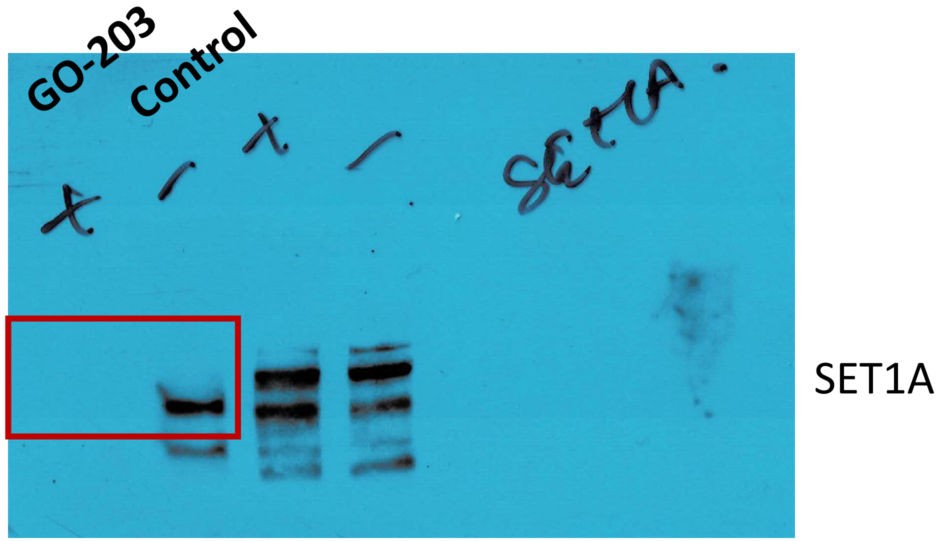

Fig. S1i

DU145 LNCaP-AI

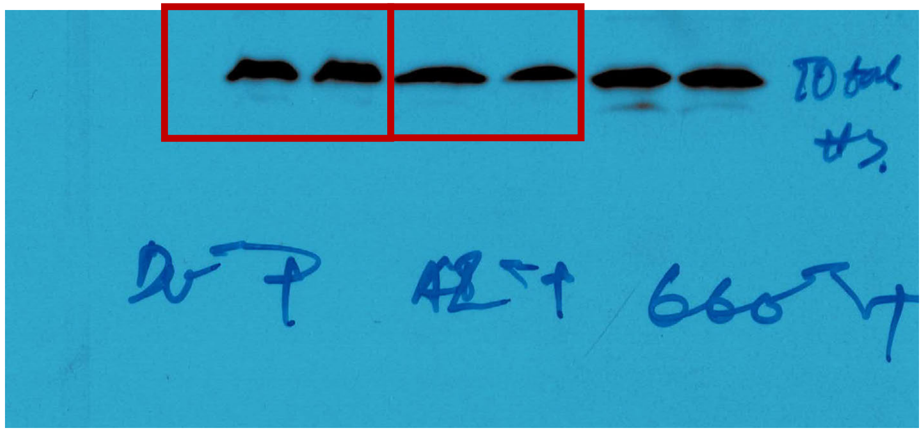

TOTAL H3

DU145 LNCaP-AI

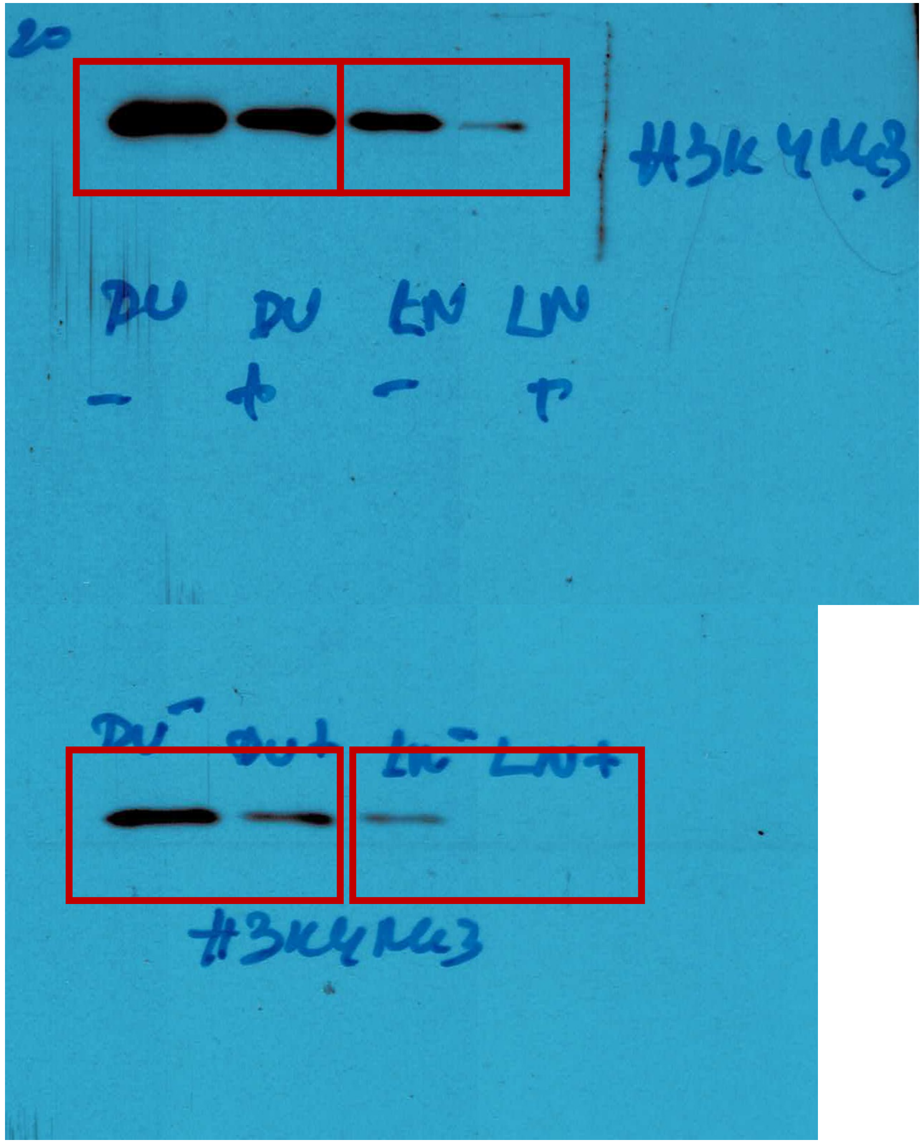

H3K4Me3

Figure 1g

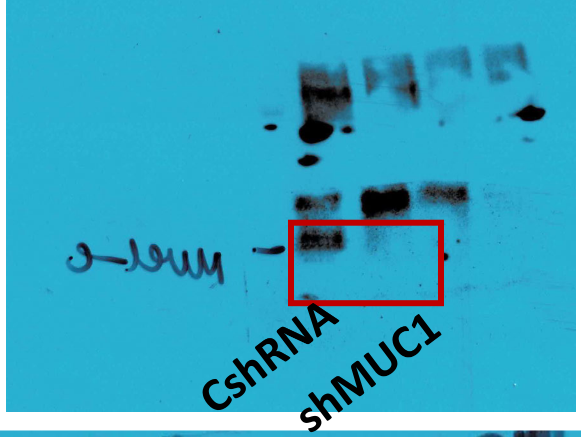

MUC1-C

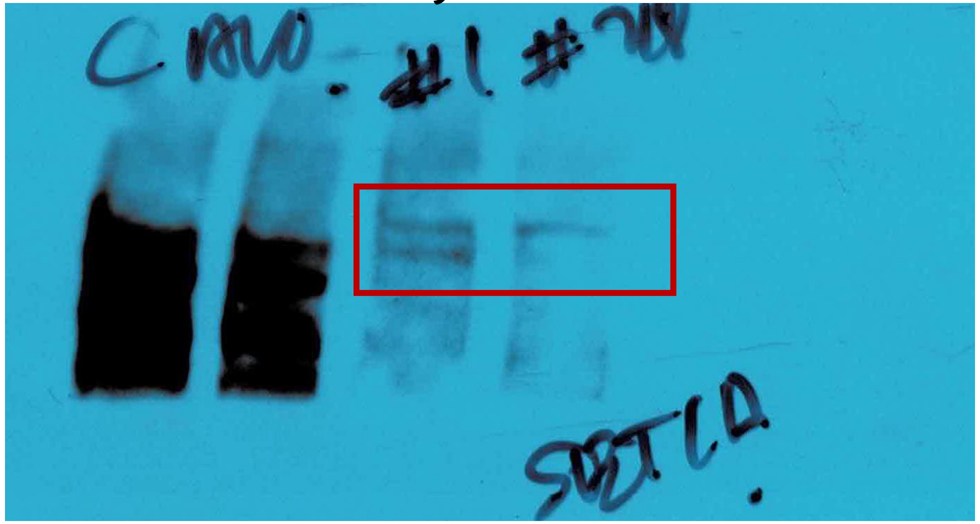

SET1A

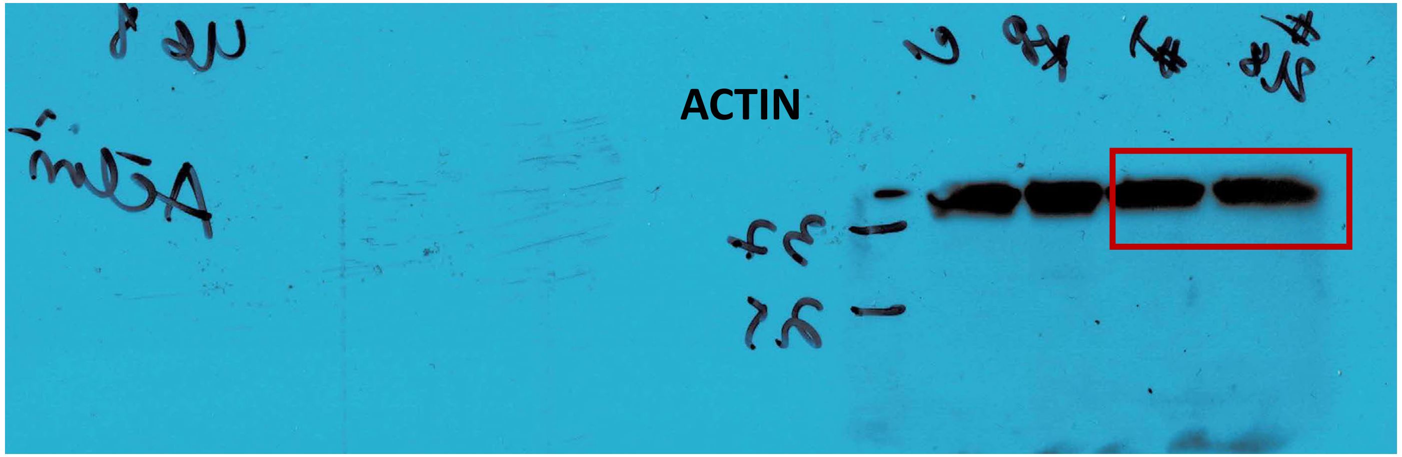

Figure S1j

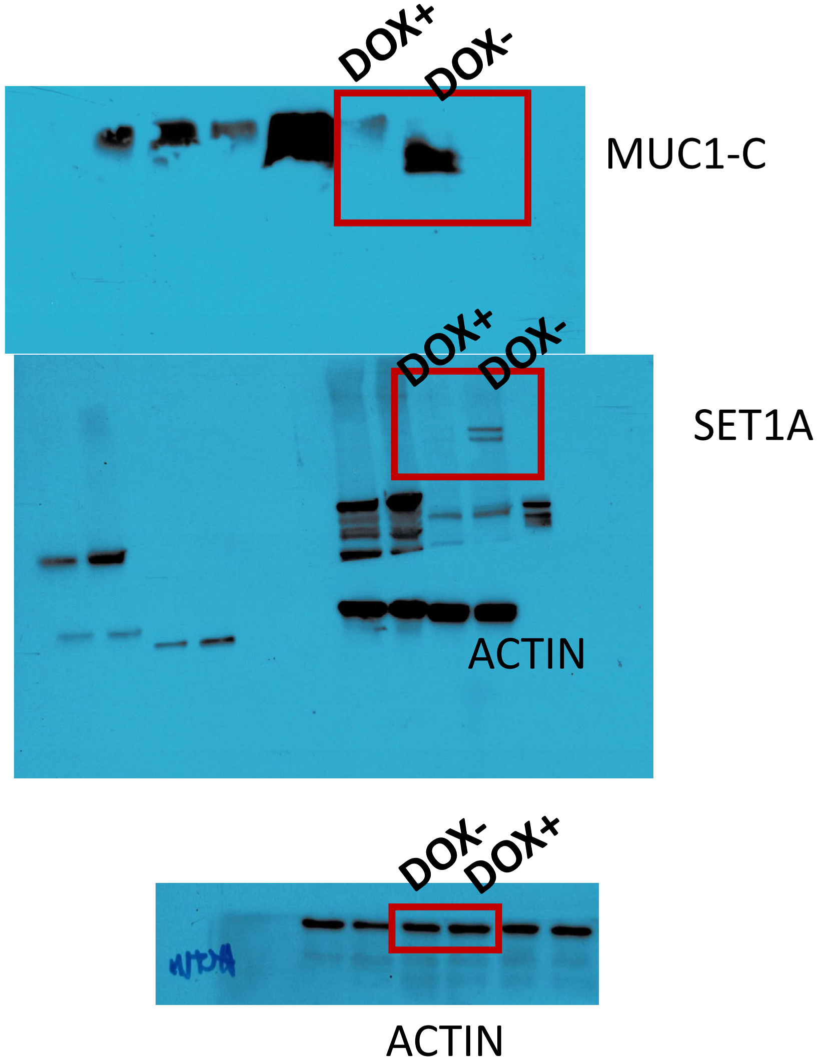

Figure S1k

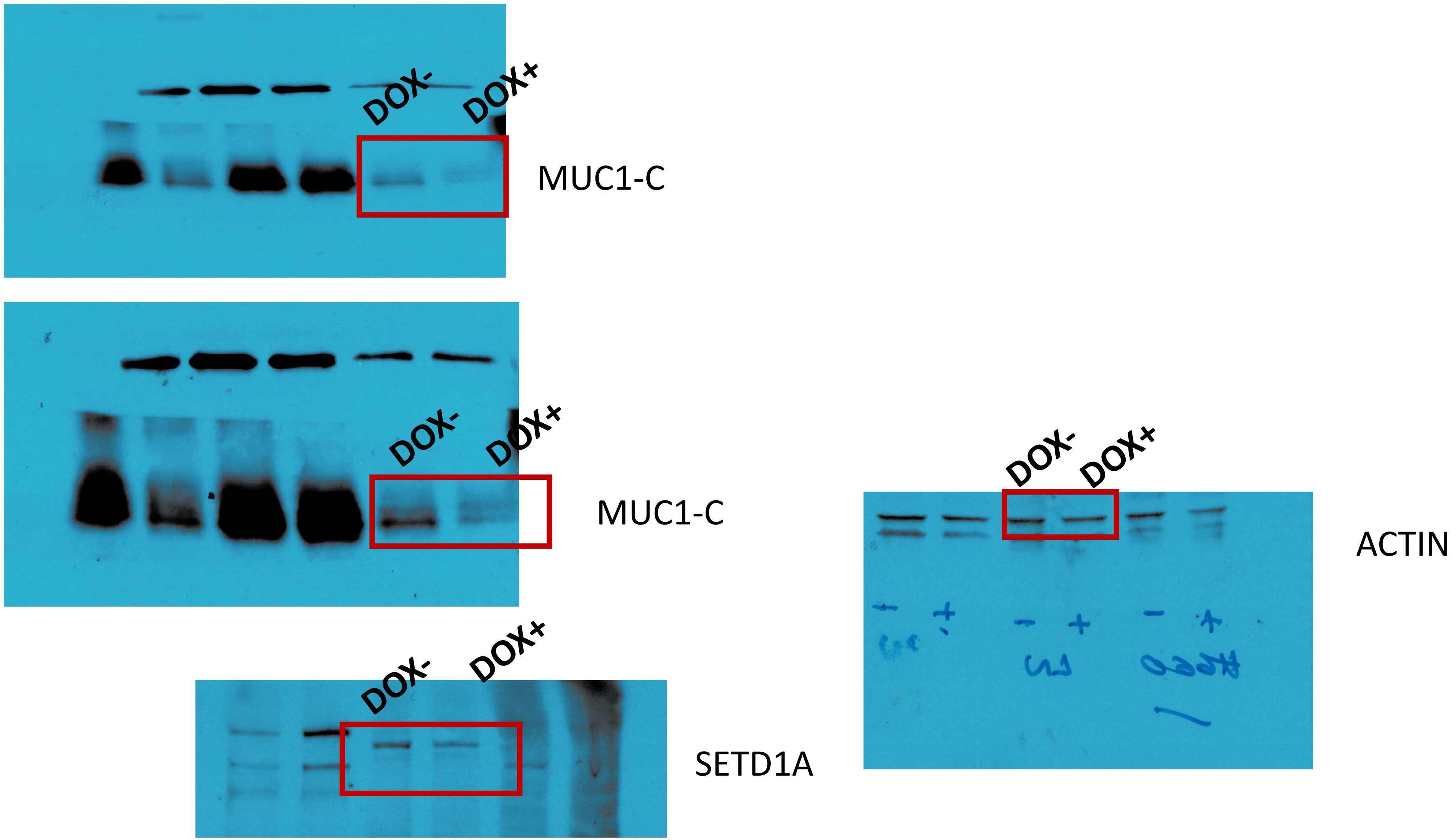

Figure S2c

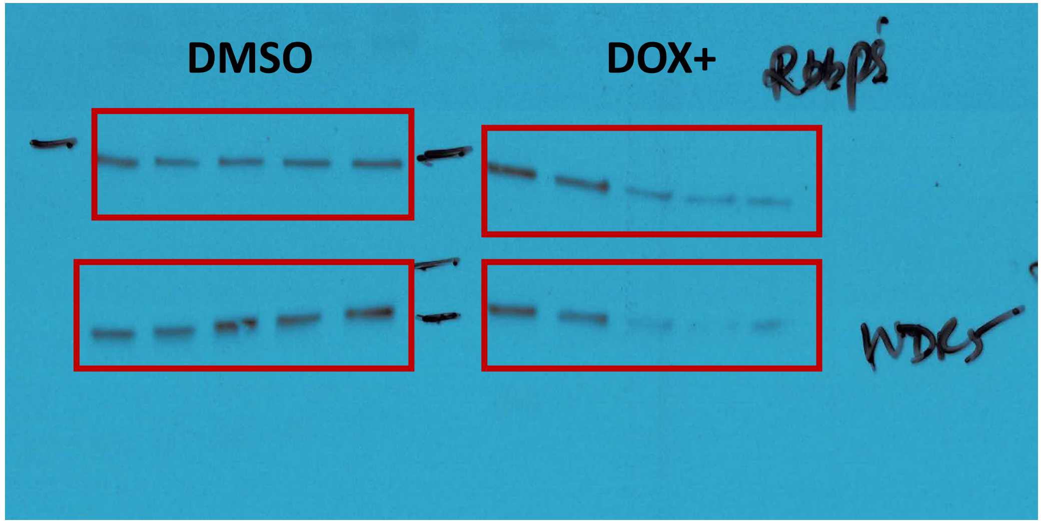

RBBP5

WDR5

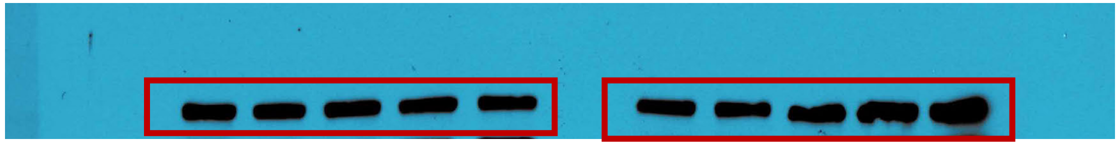

DMSO DOX+

Figure S2e

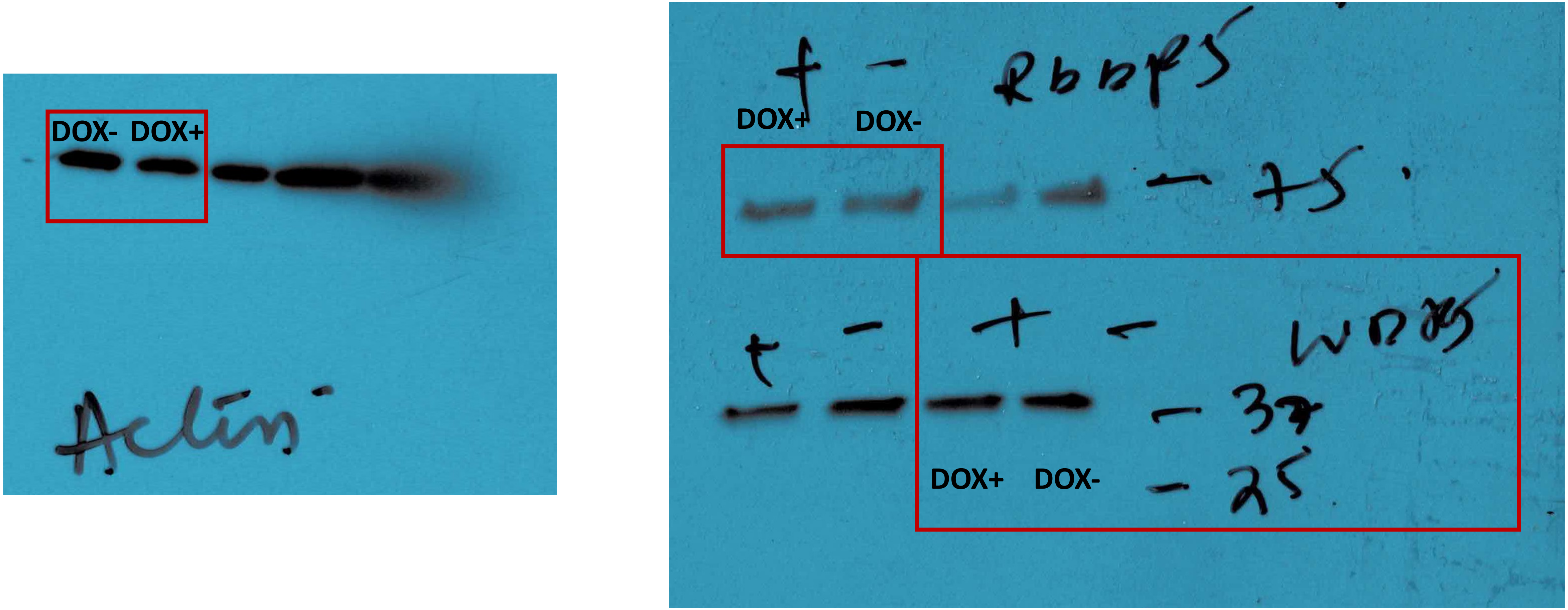

Figure S2f

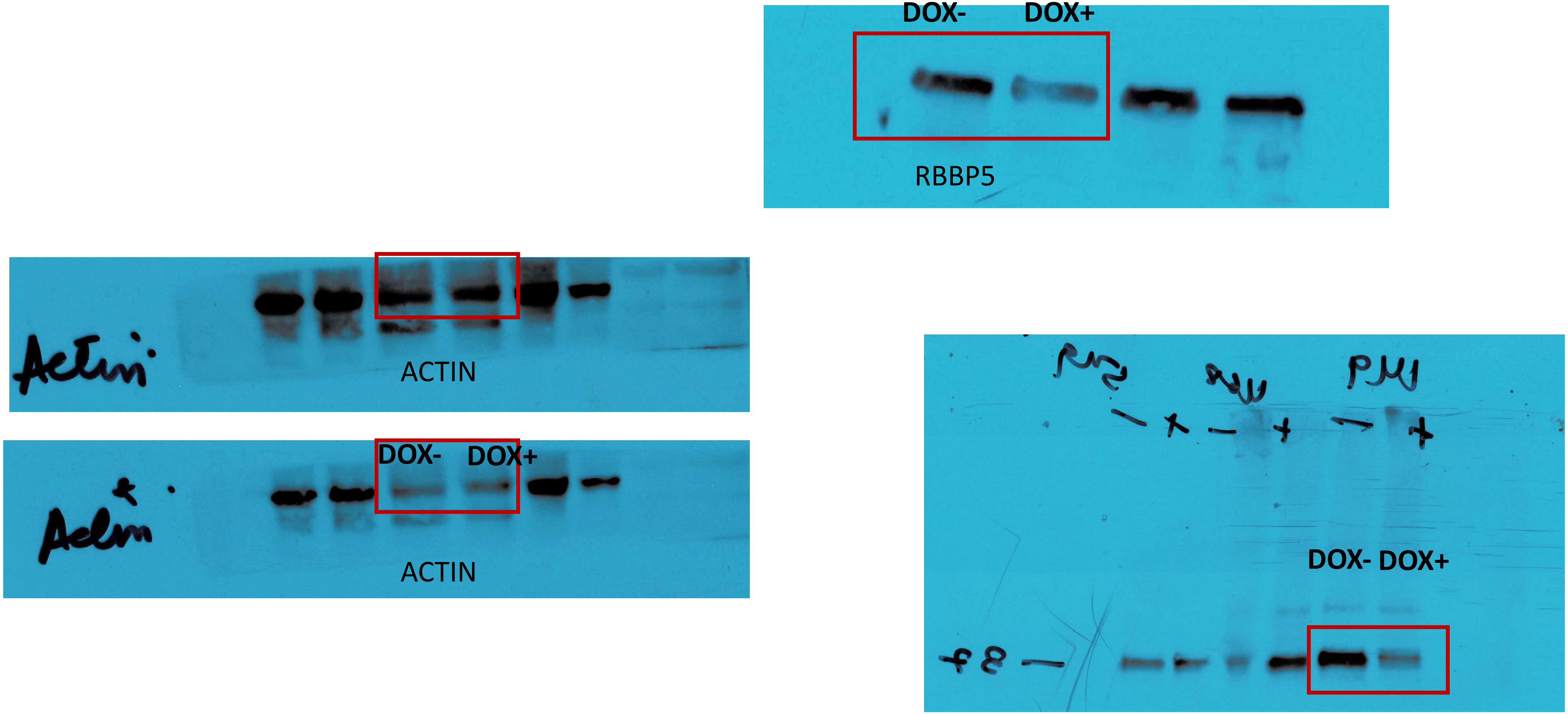

WDR5

Fig. S2g

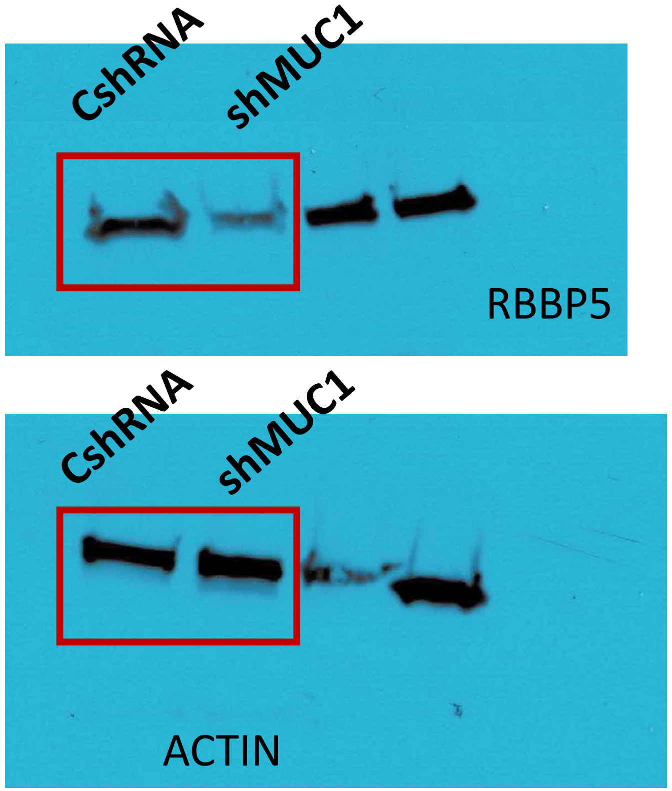

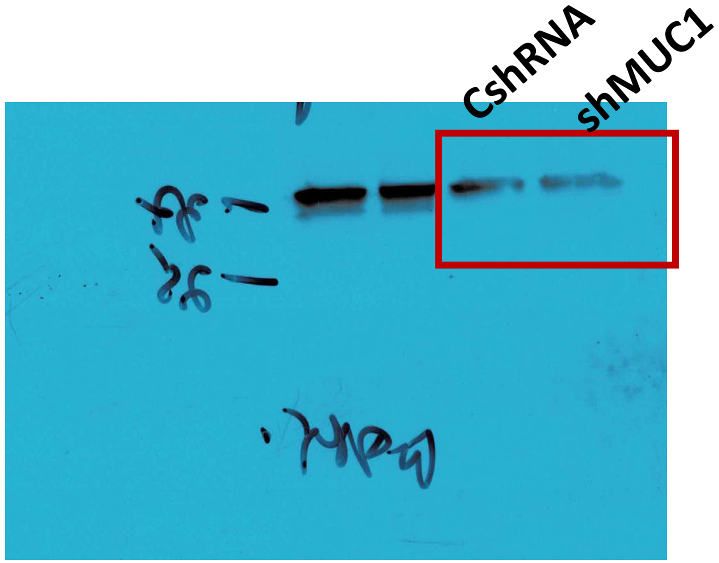

WDR5

Figure S2h

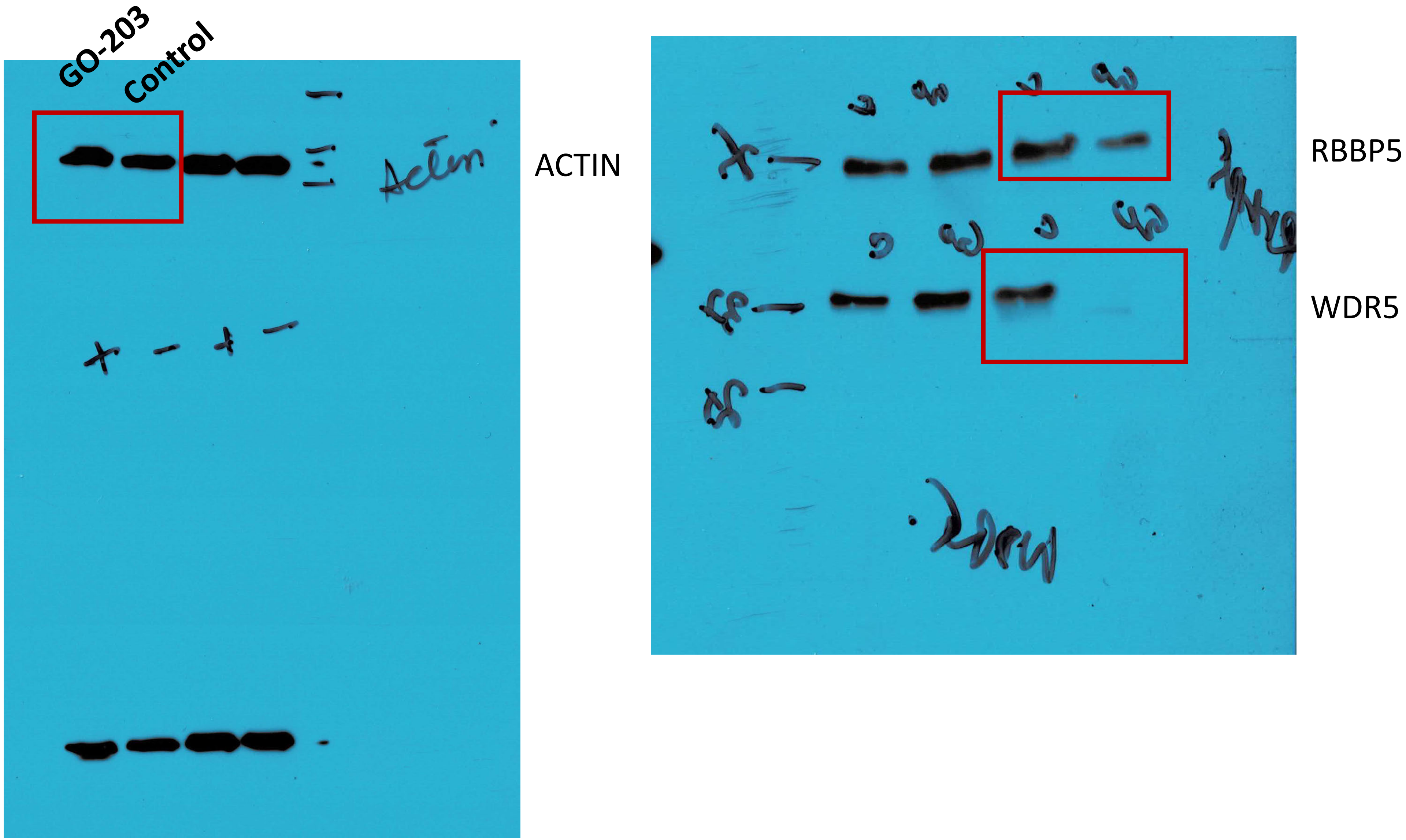

Figure S2i

ACTIN

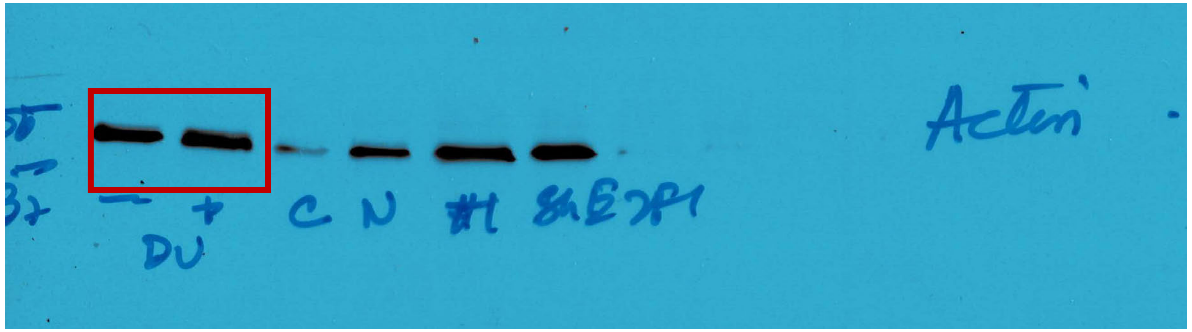

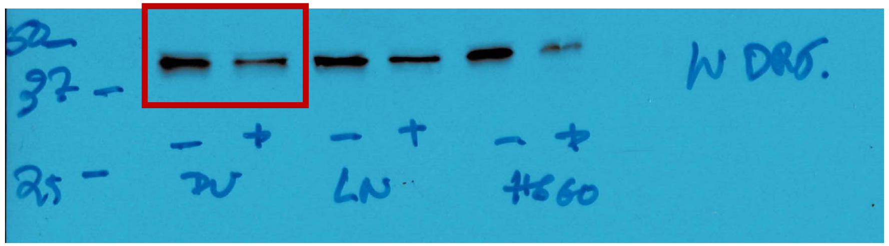

WDR5

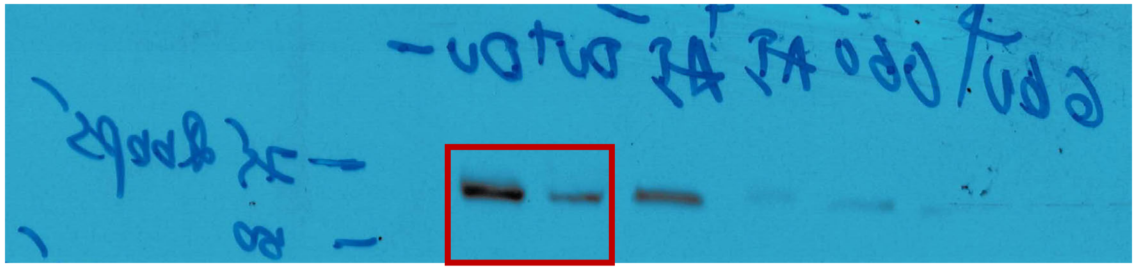

RBBP5

Figure S2j

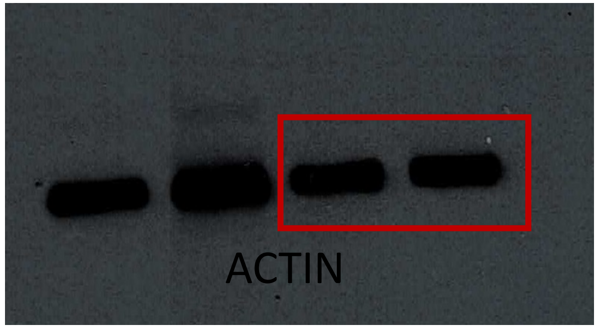

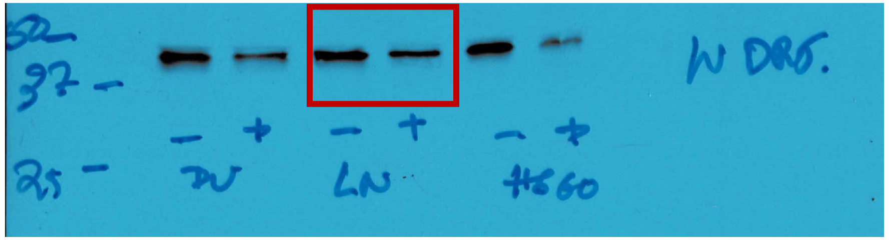

WDR5

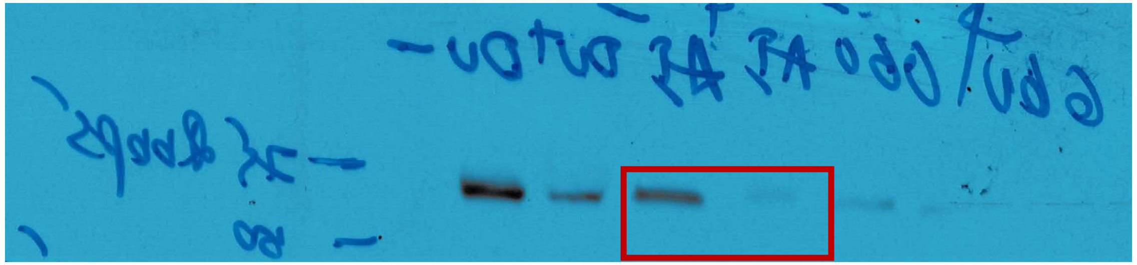

RBBP5

Figure S3a

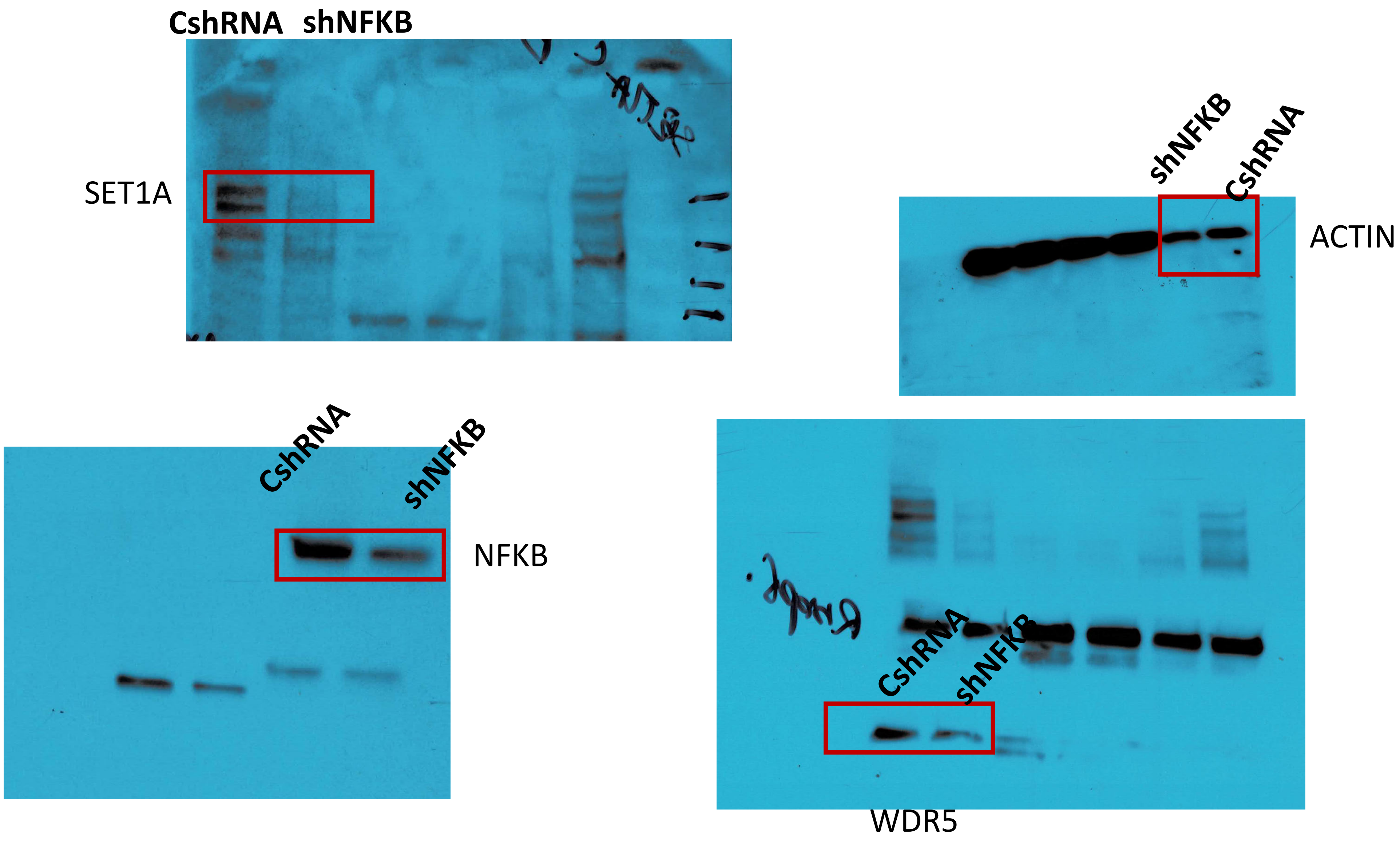
CshRNA
shNFKB
SET1A
shNFKB
CshRNA
ACTIN
CshRNA
shNFKB
NFKB
CshRNA
shNFKB
WDR5

Figure S3b

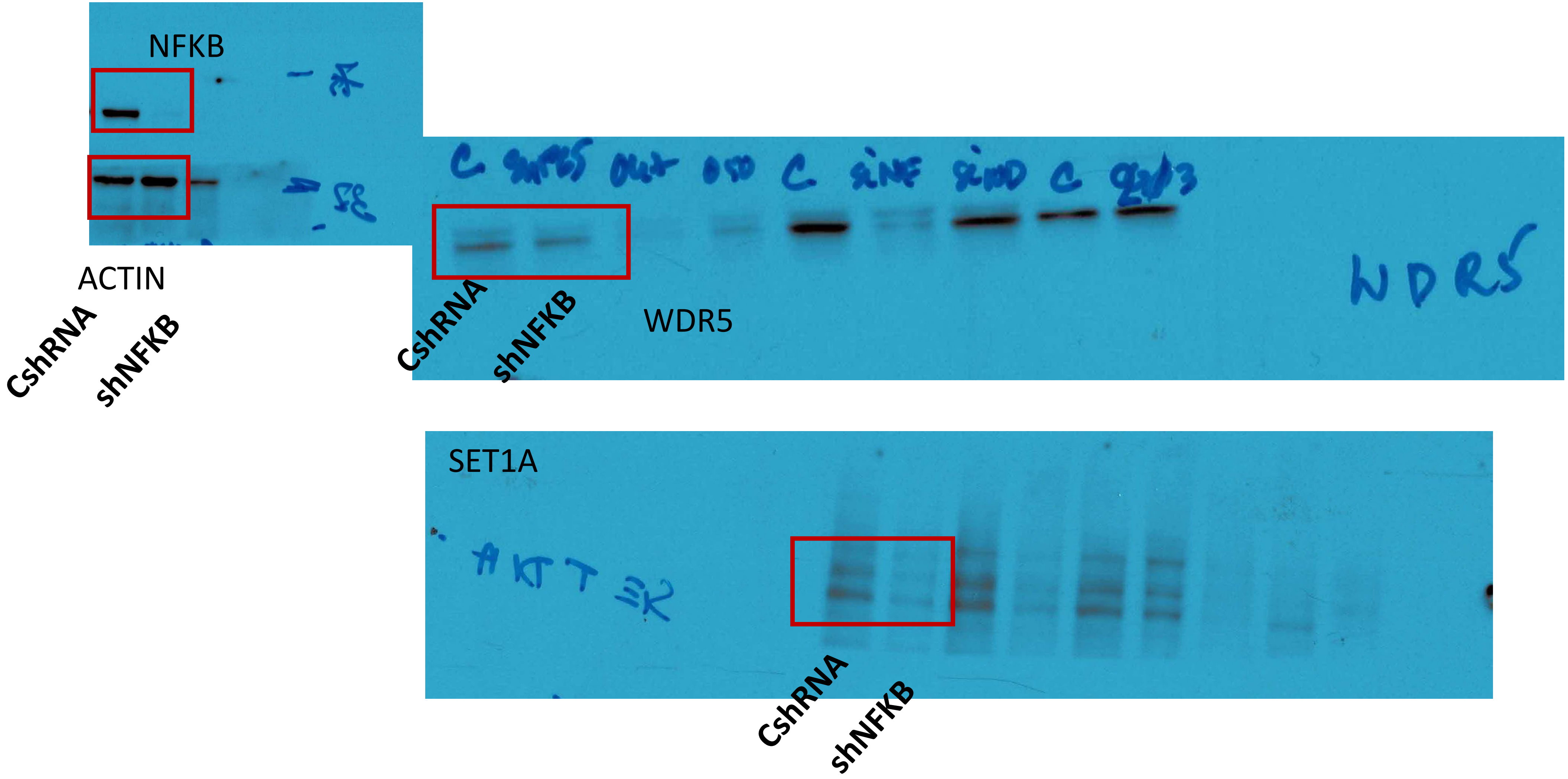
NFKB
ACTIN
CshRNA
shNFKB
WDR5
CshRNA
shNFKB
SET1A
CshRNA
shNFKB

Figure S3c

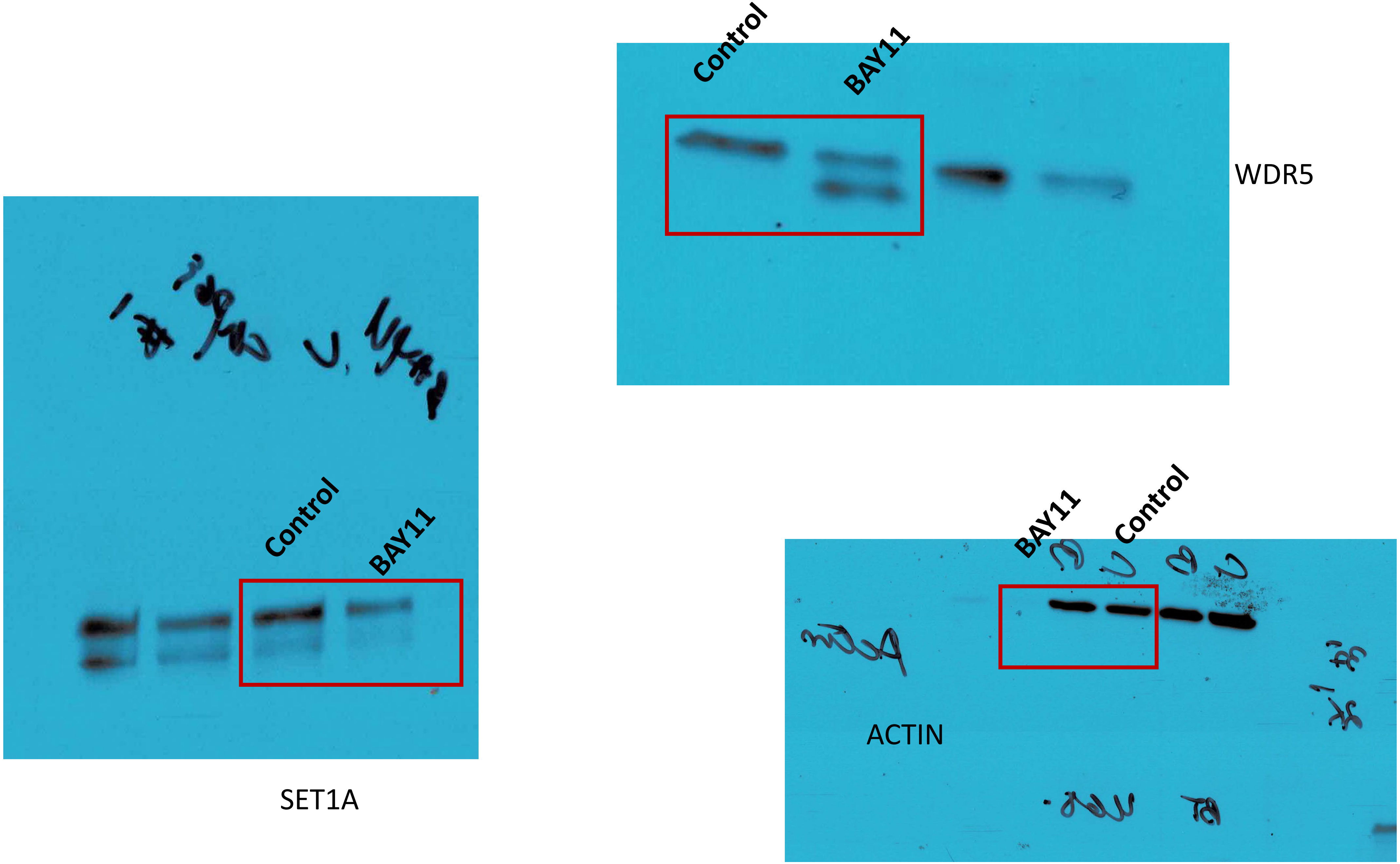
Control
BAY11
WDR5
BAY11
Control
ACTIN
Control
BAY11
SET1A

Figure S4i

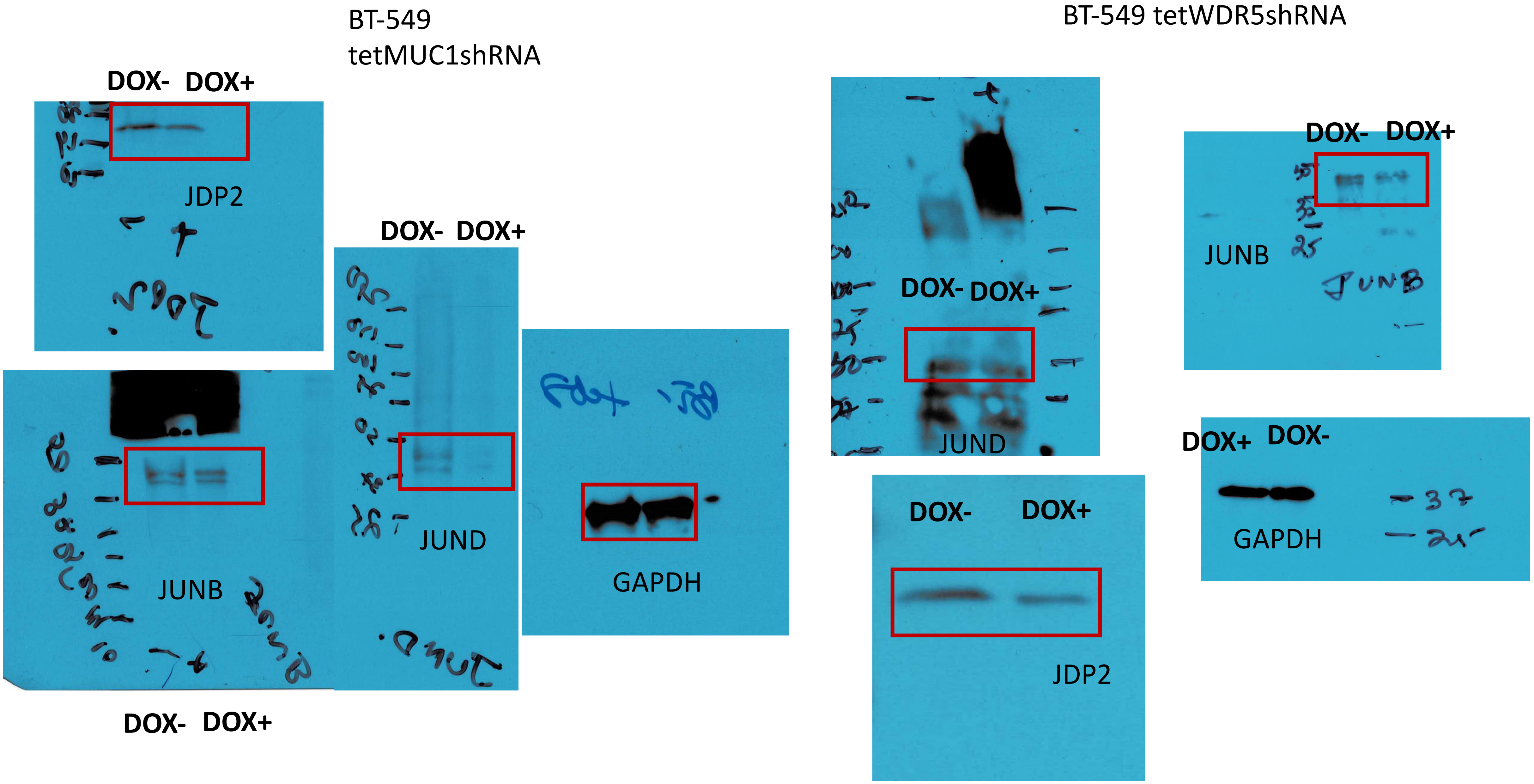
BT-549
tetMUC1shRNA
BT-549 tetWDR5shRNA
DOX- DOX+
JDP2
DOX- DOX+
JUND
GAPDH
JUNB
DOX- DOX+
DOX- DOX+
JUND
DOX- DOX+
JUNB
DOX- DOX+
JDP2
DOX+ DOX-
GAPDH

Figure S5b

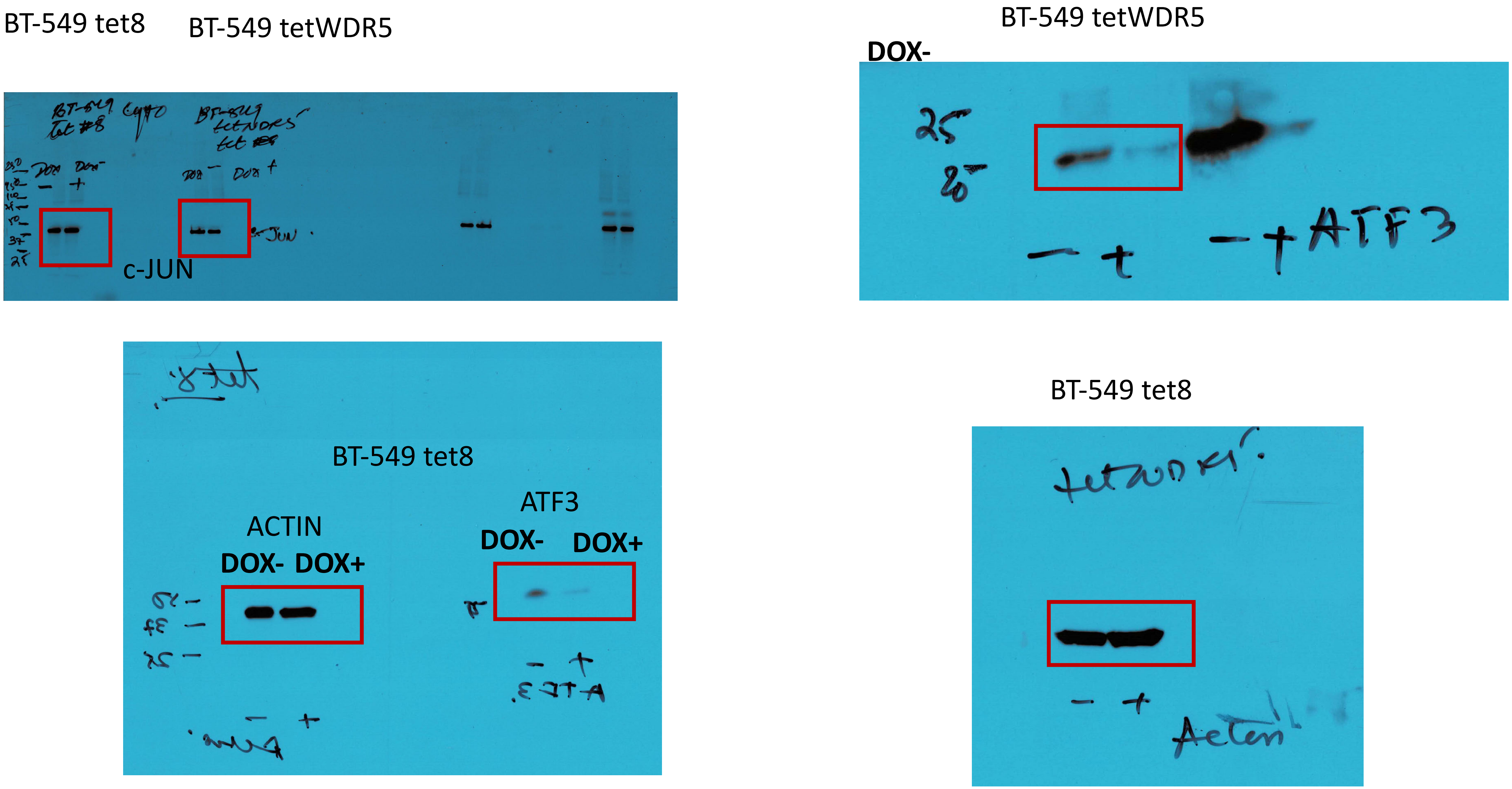
BT-549 tet8
BT-549 tetWDR5
c-JUN
BT-549 tetWDR5
DOX-
ATF3
BT-549 tet8
ACTIN
DOX- DOX+
ATF3
DOX- DOX+
BT-549 tet8
Actin
